# A general thermodynamics-triggered competitive growth model to guide the synthesis of two-dimensional nonlayered materials

**DOI:** 10.1038/s41467-023-36619-5

**Published:** 2023-02-21

**Authors:** Zijing Zhao, Zhi Fang, Xiaocang Han, Shiqi Yang, Cong Zhou, Yi Zeng, Biao Zhang, Wei Li, Zhan Wang, Ying Zhang, Jian Zhou, Jiadong Zhou, Yu Ye, Xinmei Hou, Xiaoxu Zhao, Song Gao, Yanglong Hou

**Affiliations:** 1 School of Materials Science and Engineering, Beijing Key Laboratory for Magnetoelectric Materials and Devices, Beijing Innovation Center for Engineering Science and Advanced Technology, Peking University, Beijing, 100871 China; 2 Academy for Advanced Interdisciplinary Studies, Peking University, Beijing, 100871 China; 3 State Key Laboratory for Mesoscopic Physics and Frontiers Science Center for Nano-Optoelectronics, School of Physics, Peking University, Beijing, 100871 China; 4 Center for Alloy Innovation and Design, State Key Laboratory for Mechanical Behavior of Materials, Xi’an Jiaotong University, Xi’an, 710049 China; 5 Beijing National Laboratory for Condensed Matter Physics, Institute of Physics, Chinese Academy of Sciences, Beijing, 100190 China; 6 Centre for Quantum Physics, Key Laboratory of Advanced Optoelectronic Quantum Architecture and Measurement, School of Physics, Beijing Institute of Technology, Beijing, 100081 China; 7 Innovation Research Institute for Carbon Neutrality, University of Science and Technology Beijing, Beijing, 100083 China; 8 Institute of Spin-X Science and Technology, South China University of Technology, Guangzhou, 510641 China

**Keywords:** Two-dimensional materials, Magnetic properties and materials

## Abstract

Two-dimensional (2D) nonlayered materials have recently provoked a surge of interest due to their abundant species and attractive properties with promising applications in catalysis, nanoelectronics, and spintronics. However, their 2D anisotropic growth still faces considerable challenges and lacks systematic theoretical guidance. Here, we propose a general thermodynamics-triggered competitive growth (TTCG) model providing a multivariate quantitative criterion to predict and guide 2D nonlayered materials growth. Based on this model, we design a universal hydrate-assisted chemical vapor deposition strategy for the controllable synthesis of various 2D nonlayered transition metal oxides. Four unique phases of iron oxides with distinct topological structures have also been selectively grown. More importantly, ultra-thin oxides display high-temperature magnetic ordering and large coercivity. Mn_x_Fe_y_Co_3-x-y_O_4_ alloy is also demonstrated to be a promising room-temperature magnetic semiconductor. Our work sheds light on the synthesis of 2D nonlayered materials and promotes their application for room-temperature spintronic devices.

## Abstract (web summary)

2D nonlayered materials exhibit interesting properties for catalysis, nanoelectronics and spintronics applications, but their growth is still challenging. Here, the authors report a theoretical model and an experimental strategy to synthesize various 2D nonlayered transition metal oxides with room-temperature magnetic properties.

## Introduction

Two-dimensional (2D) nonlayered materials have come under the spotlight of scientific and engineering research due to their abundant species as well as novel properties, such as enhanced surface activity^1^, anomalous thickness-dependent magnetism^2^, outstanding photodetectivity^3^, and so on, which is a crucial extension and supplement to 2D layered materials. More importantly, 2D nonlayered materials could overcome the recent dilemma faced by 2D layered magnets^4,5^, i.e., poor stability, low transition temperatures, and less variety. However, the controllable synthesis of 2D nonlayered materials remains an enormous challenge. Firstly, nonlayered materials are constructed with strong chemical bonds in all directions, inherently impeding 2D anisotropic growth. Secondly, they always have various phase structures and multiple components. For example, nonlayered iron oxides are polymorphic (Fe_3_O_4_, *γ*-Fe_2_O_3_, *ε*-Fe_2_O_3_, and *α*-Fe_2_O_3_) with disparate properties^6–9^. In this regard, selective synthesis of one single-phase crystal is very challenging, not to mention the 2D growth of multi-element nonlayered materials. Thirdly, there is still a lack of general economical methods to produce high-quality 2D single crystals with desired thickness and morphology.

In addition to experimental challenges, the understanding of 2D anisotropic growth mechanism for nonlayered materials is still in its infancy and desiderates a deep exploration. Unfortunately, major theoretical efforts are dedicated to investigating van der Waals (vdW) layered materials merely^10,11^. A generic model to interpret the growth of both layered and nonlayered materials is still missing, and quantitative models are incredibly rare. Moreover, the key driving force for forming ultra-thin 2D structures as the result of competition between vertical and lateral growth remains indistinct. The development of growth theory could not only explain the experimental phenomena but also promote the growth progress in turn. Hence, a versatile model to thoroughly comprehend 2D nonlayered material synthesis as well as the related factors is urgently desirable.

Herein, we propose a general thermodynamics-triggered competitive growth (TTCG) model to provide an in-depth understanding and powerful guidance for the synthesis of 2D nonlayered materials (including oxides, chalcogens, and oxyhalides). Guided by this model, we design a universal hydrate-assisted chemical vapor deposition (HACVD) method to synthesize a library of 2D nonlayered transition metal oxide (TMO) nanoflakes (including 9 binary, 4 ternary, and even 6 alloy oxides). Moreover, phase-controllable growth of four different iron oxides can also be achieved. Raman spectra and atomic-resolution scanning transmission electron microscopy-annular dark-field (STEM-ADF) imaging confirm the composition, phase structure, and growth orientation of as-grown nanoflakes. Importantly, various oxides exhibit attractive room-temperature magnetism, the magnetic domains of which are investigated by magnetic force microscope (MFM) measurements. Furthermore, magneto-transport characterizations reveal the room-temperature magnetic semiconductor feature of Mn_x_Fe_y_Co_3-x-y_O_4_ alloys, making them pioneering candidates for spintronic devices.

## Results

### Universal model for 2D nonlayered materials growth

**Fig. 1:**
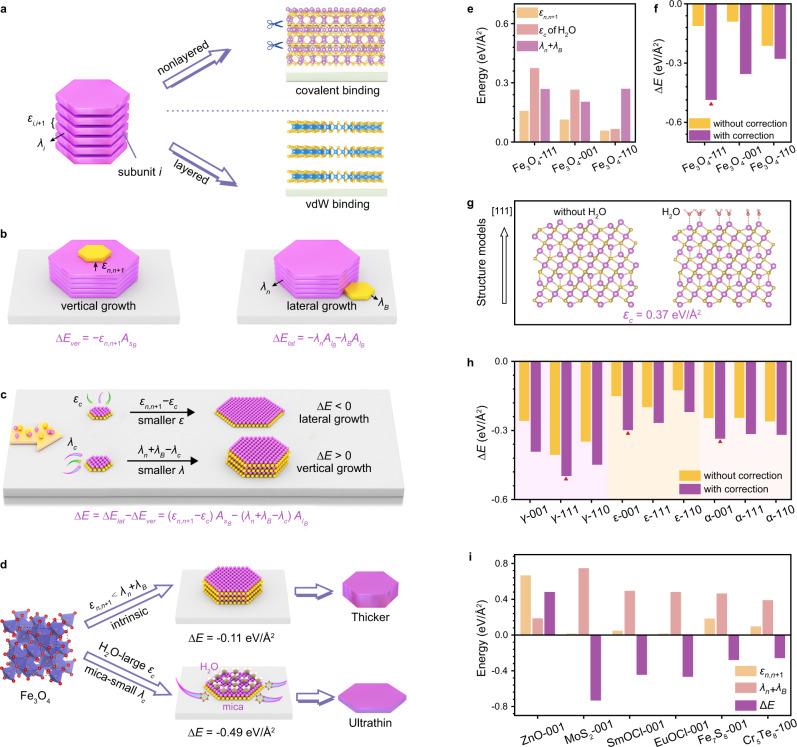
The thermodynamics-triggered competitive growth (TTCG) model. **a** Schematic illustration of ${\varepsilon }_{i,i+1}$ and ${\lambda }_{i}$ in the Eq. (1) and the representations in nonlayered and layered materials. ${\varepsilon }_{i,i+1}$ and ${\lambda }_{i}$ refer to the interaction energy per unit area between subunit *i* and *i* + 1, and the edge energy per unit area of subunit *i*, respectively. **b** Sketch map of vertical and lateral growth when a new cluster (yellow) grows with the *n* stacking subunits (lilac). The free energy change of vertical growth mainly comes from the interaction of subunit at the upper interface, while edge energy penalty is dominant for lateral growth. ∆*E*_*ver*_ and ∆*E*_*lat*_ represent the free energy change of vertical and lateral growth. ${\varepsilon }_{n,n+1}$ denotes the interaction energy between subunit *n* and *n* + 1. ${\lambda }_{n}$ and ${\lambda }_{B}$ represent the average edge energies of the initial structure and the new cluster, respectively. ${A}_{{s}_{B}}$ and ${A}_{{l}_{B}}$ are the basal and lateral contact areas. **c** The competition between $\varepsilon$ term and $\lambda$ term. Smaller $\varepsilon$ term will promote lateral growth and smaller $\lambda$ term leads to vertical growth. ${\varepsilon }_{c}$ is the circumstance correction term of the interaction energy and ${\lambda }_{c}$ is the circumstance correction term of the edge energy. **d** A schematic for the growth process of 2D Fe_3_O_4_ nanoflakes along the [111] direction guided by the TTCG model. The intrinsic binding energy difference is not negative enough. The facilitation of H_2_O passivation (large ${\varepsilon }_{c}$) and mica substrate (small ${\lambda }_{c}$) lead to smaller ∆*E*, and therefore promote the synthesis of ultrathin Fe_3_O_4_ nanoflakes. **e** Every calculated value in the model for Fe_3_O_4_ with different orientations. **f** Total ∆*E* with and without circumstance correction terms for Fe_3_O_4_ with different orientations. **g** Optimized structures of Fe_3_O_4_ along the [111] direction without and with the H_2_O adsorption. **h** Total ∆*E* of *γ*-, *ε*-, *α*-Fe_2_O_3_ in different orientations with and without correction terms. Red triangles indicate the lowest energy. **i** Calculated values in the growth model of ZnO, MoS_2_, SmOCl, EuOCl, Fe_7_S_8_, and Cr_5_Te_8_.

At first, we put forward a common formula to describe the energy of a material system. As shown in Fig. 1a, we assumed all materials can be regarded as *n* superimposed subunits (a slab with the smallest repeating unit along the *z*-axis) that are interconnected to each other. The total free energy of the system (*E*_*free*_) with *n* superimposed subunits consists of the following parts: (1) the energy of every stacking subunit that exists alone, (2) interunit interaction, (3) edge formation energy. Hence, *E*_*free*_ can be expressed as

1 $${E}_{{free}}=\mathop{\sum }\limits_{i=1}^{n}{{E}_{i}A}_{S}-\mathop{\sum }\limits_{i=1}^{n-1}{\varepsilon }_{i,i+1}{A}_{S}+\mathop{\sum }\limits_{i=1}^{n}{\lambda }_{i}{A}_{L}$$

where *E*_*i*_, *ε*_*i,i*+1_, *λ*_*i*_ refer to the free energy per unit area of isolated subunit *i*, the interaction energy between subunit *i* and *i* + 1, and the edge energy of subunit *i*, respectively. *A*_*S*_ and *A*_*L*_ denote the surface and lateral area.

In layered materials, a subunit is generally represented by one slab between adjacent gaps, and weak vdW interaction plays a leading role between stacking subunits. As for nonlayered materials (Supplementary Figs. 1–3), the selection of subunit is more complex and needs to be discussed individually. More importantly, non-negligible strong covalent binding plays a part, so *ε*_*i,i*+1_ is relatively larger and influenced by *n*. A detailed comparison is shown in Supplementary Table 1.

Chemical vapor deposition (CVD) method is considered as a facile route to obtain various high-quality 2D materials^12,13^. In the CVD growth process, we assume a new growth cluster (with the basal and lateral contact area of ${A}_{{s}_{B}}$ and ${A}_{{l}_{B}}$, which are supposed to be equal) combines with the initial structure (*n* stacking subunits) in two ways, i.e., vertically or laterally (Fig. 1b). Based on the above analysis (more details in Supplementary Note 1), the free energy change of vertical growth (∆*E*_*ver*_) mainly comes from the binding force of subunit at the upper interface, while edge energy penalty is dominant for lateral growth (∆*E*_*lat*_):

2 $${\triangle E}_{{ver}}=-{\varepsilon }_{n,n+1}{A}_{{s}_{B}}$$

3 $${\triangle E}_{{lat}}=-{\lambda }_{n}{A}_{{l}_{B}}-{\lambda }_{B}{A}_{{l}_{B}}$$

where *λ*_*n*_ and *λ*_*B*_ represent the average edge energies of the initial structure and the new cluster, respectively. The values of ${\triangle E}_{{ver}}$ and ${\triangle E}_{{lat}}$ directly reflect the growth tendency along vertical or lateral direction, so their difference (∆*E*) can be evaluated as the criterion of growth modes:

4 $${\triangle E=\triangle E}_{{lat}}-{\triangle E}_{{ver}}={\varepsilon }_{n,n+1}{A}_{{s}_{B}}-\left({\lambda }_{n}+{\lambda }_{B}\right){A}_{{l}_{B}}$$

In fact, surrounding microscopic atomic circumstances could also influence these energies in a realistic growth process, in addition to the material itself. Consequently, we introduce the circumstance correction terms (${\varepsilon }_{c}$ and ${\lambda }_{c}$) to optimize our model, and the Eq. (4) can be modified as follows:

5 $$\triangle E=({\varepsilon }_{n,n+1} - {\varepsilon }_{c})A_{{s}_{B}}-\left({\lambda }_{n}+{\lambda }_{B}-{\lambda }_{c}\right){A}_{{l}_{B}}$$

Accordingly, lateral or vertical growth is the result of competition between $\varepsilon$ term (${\varepsilon }_{n,n+1}-{\varepsilon }_{c}$) and $\lambda$ term (${\lambda }_{n}+{\lambda }_{B}-{\lambda }_{c}$), which is based on thermodynamics triggering (Fig. 1c). 2D growth (lateral growth configuration) is favored when $\triangle E$ is negative. With respect to the intrinsic features of materials, a larger difference between ${\varepsilon }_{n,n+1}$ and ${\lambda }_{n}$ (${\varepsilon }_{n,n+1}$ is smaller and ${\lambda }_{n}$ is larger) will promote 2D growth. For instance, ${\varepsilon }_{n,n+1}$ is rather small in vdW materials, consistent with their monolayer growth tendency^14,15^. Moreover, the interface adsorption or passivation can increase ${\varepsilon }_{c}$ (by interacting with surface atoms) to decrease $\triangle E$ and facilitate 2D growth^16,17^, which has not been quantitatively exhibited in previous literatures. Furthermore, substrates are also of great importance by influencing *λ*_*c*_, as the diffusion barrier resists edge growth^18^, which can be equivalent to an additional edge energy correction (Supplementary Table 2).

In brief, as-proposed model provides a concrete criterion to predict and guide 2D growth, including the effect of both intrinsic materials characteristics and exterior growth conditions. Besides, it offers a quantitative way to predict the growth modes (vertically or laterally) or 2D growth difficulty based on the sign and absolute value of ∆*E*. More importantly, the key growth factor is clarified by analyzing the influence of multivariate, providing powerful guidance for effective regulation of experiments. This model is applicable both to layered and especially to nonlayered materials, extending the theoretical research on 2D materials growth.

### Synthesis of 2D nonlayered materials guided by TTCG model

TMO is a crucial class of nonlayered family with unique magnetic properties, such as topological spin states^19,20^, multiferroic order^21,22^, and Verwey transition^23^, which have held great prospects in spin filter^24,25^, magnetic recording^26^, magnetoelectric coupling^27,28^, and so on. The formation of ultrathin TMO may overcome the limitations of 2D magnets, but its controllable growth is still a grand difficulty. Therefore, we focus on TMO as the main examples to illustrate the TTCG model.

Cubic Fe_3_O_4_ is taken as a representative example, because various oxides have similar structures (Supplementary Table 3). The intrinsic energy difference between ${\varepsilon }_{n,n+1}$ and ${\lambda }_{n}$+${\lambda }_{B}$ along diverse orientations is not negative enough (Fig. 1d–f, and Supplementary Table 4), so the inherent 2D growth is thermodynamically challenging. According to Eq. (5), increasing ${\varepsilon }_{{{{{{\rm{c}}}}}}}$ to reduce $\varepsilon$ term is an effective approach for promoting lateral growth. Considering the surface metal atoms (Supplementary Fig. 1e), adsorption of polar water molecules is found to significantly influence ${\varepsilon }_{c}$ (Fig. 1e and Supplementary Table 4). For instance, ${\varepsilon }_{c}$ of water is as large as 0.37 eV/Å^2^ for Fe_3_O_4_ along the [111] direction (Fig. 1g). Besides, enhancing *λ* term is another way to decrease ∆*E*, so mica with smaller diffusion barrier energy (i.e., smaller ${\lambda }_{c}$) is considered as a better growth substrate (Supplementary Table 2). As a result, [111] direction is predicted to be the preferred growth orientation with the lowest ∆*E* of −0.49 eV/Å^2^ (Fig. 1f), which is more negative at this time to favor 2D growth (Fig. 1d). In addition to Fe_3_O_4_, other Fe-based oxides are also predicted to have a good chance to form 2D nanoflakes under the action of water and mica (Fig. 1h), which are thoroughly discussed in the next section.

**Fig. 2:**
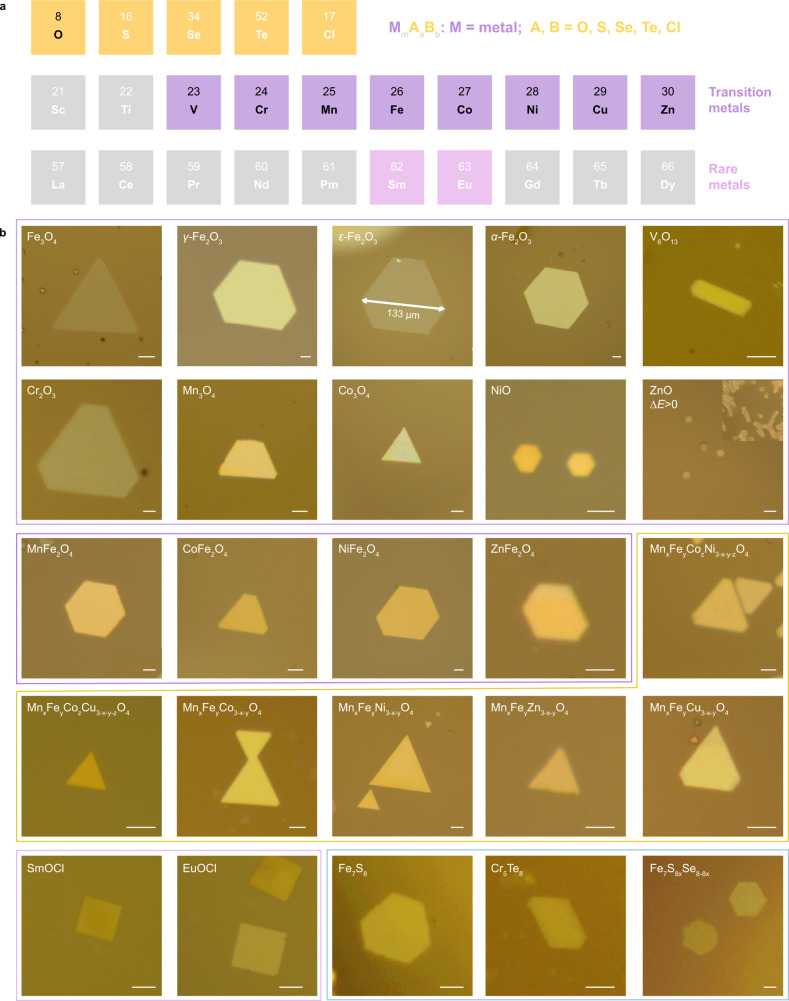
Synthesis of nonlayered materials guided by TTCG model. **a** Summary of the nonlayered materials applicable to the TTCG model. The elements highlighted in purple or pink denote transition or rare metals whose oxides, oxyhalides, or chalcogenides with nonlayered structures are demonstrated to have the potential to form 2D structures. The elements highlighted in yellow represent the available anions of nonlayered materials. The elements in black color indicate their oxides can be synthesized by our hydrate-assisted chemical vapor deposition (HACVD) method. m, a, and b are the corresponding stoichiometric numbers. **b** Optical images of as-grown materials. Scale bars, 5 μm. Binary, ternary, and alloy oxides are outlined in lilac, purple, and yellow colors, respectively. Rare earth oxyhalides are outlined in pink and transition metal chalcogens are marked in blue.

Based on these theoretical analyses, a HACVD growth strategy was designed to produce TMO nanoflakes on mica substrate. Hydrate is adopted to deliver a controlled amount of water vapor at a preset time to promote 2D growth. Transition metal chloride with lower melting points and oxygen were employed as precursors. Several ultra-thin 2D oxides can be synthesized by this method, including iron oxides, V_6_O_13_, Cr_2_O_3_, Mn_3_O_4_, Co_3_O_4_, and NiO nanoflakes, as indicated in Fig. 2. However, ZnO nanorods are obtained instead of nanoflakes. The calculated Δ*E* of ZnO based on the model is positive with 0.48 eV/Å^2^ (Fig. 1i), so it is unfavorable for 2D growth, which is in good agreement with the experiment. In addition, because chlorides are employed as precursors whose melting points are close among different metals, the HACVD method can also be extended to multi-element oxides. The details are discussed in the following. Four types of ternary ferrite MFe_2_O_4_ (M = Mn, Co, Ni, Zn) and six kinds of alloy oxides (quaternary and quinary oxides) are obtained. The thickness of these 2D oxides can be down to several nanometers (Supplementary Fig. 4), thanks to the use of H_2_O and mica under the guidance of the model.

In addition to oxides, we also apply this model to other layered and nonlayered materials (Fig. 1i and Supplementary Table 4). As is well-known, layered MoS_2_ is inclined to grow into 2D monolayer^29,30^, in line with the calculated ∆*E* (as low as −0.73 eV/Å^2^). Besides, some nonlayered rare-earth metal oxyhalides (EuOCl, SmOCl) and transition metal chalcogens (Cr_5_Te_8_, Fe_7_S_8_, and In_2_Se_3_) are also predicted to possess intrinsic negative ∆*E* with a good potential to form 2D nanoflakes, which is experimentally verified by CVD method as well (Fig. 2b). The synthesis of these binary and even multi-element nonlayered oxides, chalcogens, and oxyhalides offer credible evidence for the universality and feasibility of our growth model.

### Demonstration of TTCG model in oxides of distinct structures

**Fig. 3:**
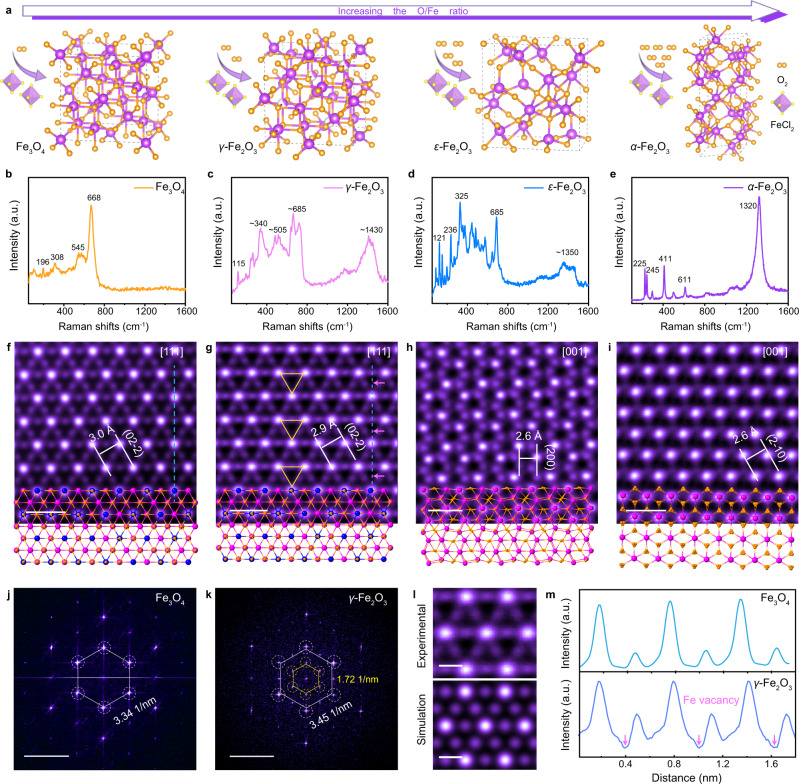
Structural characterizations of four iron oxides with different structures. **a** Structures of four iron oxides. With increasing the O/Fe ratio, Fe_3_O_4_, *γ*-Fe_2_O_3_, *ε*-Fe_2_O_3_, and *α*-Fe_2_O_3_ in different structures can be obtained, respectively. Double spheres represent O_2_ molecules; octahedrons represent FeCl_2_ molecules; the orange and purple spheres denote O and Fe atoms, respectively. Dashed rectangles represent unit cells. **b**–**e** Raman vibration modes are indexed to Fe_3_O_4_ (**b**), *γ*-Fe_2_O_3_ (**c**), *ε*-Fe_2_O_3_ (**d**), and *α*-Fe_2_O_3_ (**e**), respectively. Raman spectra of four iron oxides are totally different, and the main peaks are indicated in the figures. **f**–**i** Top-view scanning transmission electron microscopy-annular dark-field (STEM-ADF) images of Fe_3_O_4_ (**f**), *γ*-Fe_2_O_3_ (**g**), *ε*-Fe_2_O_3_ (**h**), and *α*-Fe_2_O_3_ (**i**), respectively, inserted with projected atomic models. The periodic yellow triangles indicate ordered Fe octahedral vacancies. The orange, purple, and blue spheres represent O, octahedral Fe, and tetrahedral Fe atoms, respectively. **j**, **k** The corresponding fast Fourier transform (FFT) patterns of Fe_3_O_4_ (**j**) and *γ*-Fe_2_O_3_ (**k**) along the [111] direction. **l** The enlarged STEM image of *γ*-Fe_2_O_3_ and its corresponding simulated image. **m** The corresponding intensity line profiles from Fe_3_O_4_ and *γ*-Fe_2_O_3_. The arrows indicate Fe vacancies. Scale bars: **f**–**i** 0.5 nm; **j**, **k** 1/5 nm; **l** 0.2 nm.

Notably, iron oxides possess diversified structures or phases in different space groups (Fig. 3a and Supplementary Table 3). Based on the TTCG model (Fig. 1h and Supplementary Table 4), cubic *γ*-Fe_2_O_3_, orthogonal *ε*-Fe_2_O_3_, and trigonal *α*-Fe_2_O_3_ also possess relatively negative ∆*E* (−0.50 eV/Å^2^, −0.30 eV/Å^2^, −0.34 eV/Å^2^), after introducing the correction term of water (${\varepsilon }_{c}$), indicating that they all have the potential to form 2D structures with the predicted orientation along the [111], [001], and [001] direction, respectively.

Indeed, four kinds of ultrathin iron oxides nanoflakes with distinct topology structures can be synthesized by the HACVD method. The thickness can also be regulated by changing the mass of hydrate (Supplementary Fig. 5), illustrating the importance of ${\varepsilon }_{c}$ by water for promoting 2D growth. These results verify the reliability of the prediction results of the TTCG model. Moreover, the phase-controllable growth can be realized as well, which is found to strongly depend on the O/Fe ratio in HACVD process (Supplementary Fig. 6 and Supplementary Table 5). As exhibited in Fig. 3b–e and Supplementary Table 6, four kinds of iron oxides exhibit entirely different Raman vibration modes, making them easily distinguished^9,31–36^.

To further study the atomic structure and growth orientation of the iron oxides, atomic-resolution STEM-ADF imaging was employed to reveal the structures of diverse phases. Fe atoms can be directly distinguished from O atoms by the bright contrast. STEM-ADF images of Fe_3_O_4_ and *γ*-Fe_2_O_3_ (Fig. 3f, g) reveal a periodic bright and dark spots arrangement, i.e., each bright atomic column is surrounded by dark hexagon-arranged atomic columns, which is the hallmark of approximately isostructural cubic Fe_3_O_4_ and *γ*-Fe_2_O_3_. Based on their structures, the bright contrast is mainly related to the quantity of Fe atoms rather than the atomic number. Specifically, the light dots are atomic columns containing both tetrahedral and octahedral sites, while the dark dots only contain octahedral sites, consistent with the projected atomic structure along [111] direction. Intriguingly, the fast Fourier transform (FFT) image of *γ*-Fe_2_O_3_ (Fig. 3k) exhibits a series of periodic superspots (marked by the yellow circles), which is absent in Fe_3_O_4_ (Fig. 3j). The superspots are originated from ordered Fe octahedral vacancies, labeled in Fig. 3g and Fig. 3m, in line with the simulated images (Fig. 3l). Although the presence of ordered vacancies in *γ*-Fe_2_O_3_ crystals was known from previous works^37,38^, the STEM image presented here provides the real-space evidence for octahedral vacancies in *γ*-Fe_2_O_3_. The structures of orthorhombic *ε*-Fe_2_O_3_ and trigonal *α*-Fe_2_O_3_ are also verified by the consistency of the experimental images and the atomic models along [001] zone axes, respectively (Fig. 3h, i). Notably, the observed crystal orientations of STEM are all consistent with the predictions of the model.

The successful growth of 2D nonlayered iron oxides with four different structures demonstrates TTCG model is not limited by the crystal structures, illustrating the universality of our model from another perspective. It is worth noting that although Fe_3_O_4_, *γ*-Fe_2_O_3_, and *ε*-Fe_2_O_3_ have been prepared by CVD method previously^34–36^, the key factor of phase-controllable growth is revealed here. The sizes and qualities of our samples also exceeded the preceding reports (compared in Supplementary Table 7).

### Demonstration of TTCG model in multi-element oxides

**Fig. 4:**
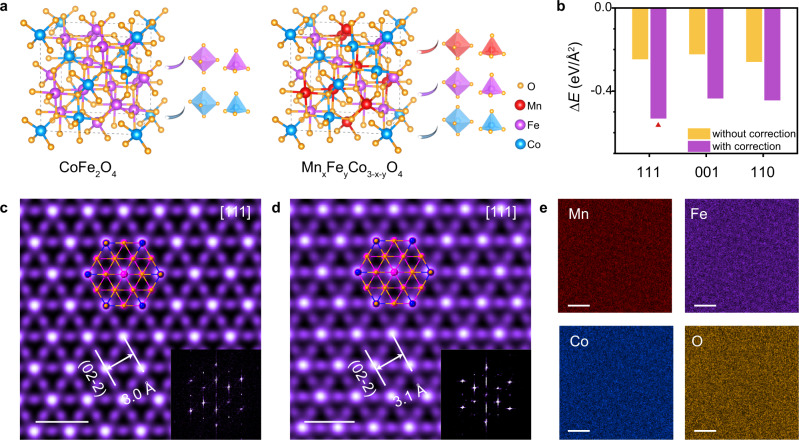
TTCG model for multi-element oxides. **a** Structure models of CoFe_2_O_4_ and Mn_x_Fe_y_Co_3-x-y_O_4_. Metal ions occupy the centers of tetrahedrons or octahedrons sites surrounded by oxygen atoms. Dashed rectangles represent unit cells. **b** Total ∆*E* with and without circumstance correction terms for CoFe_2_O_4_ with different orientations based on the TTCG model. Red triangles indicate the lowest energy. **c**, **d** Top-view STEM-ADF images of CoFe_2_O_4_ (**c**) and Mn_x_Fe_y_Co_3-x-y_O_4_ (**d**) nanoflakes, inserted with corresponding projected atomic models along the [111] orientation. The orange, purple, and blue spheres represent O atoms, octahedral sites, and tetrahedral sites, respectively. Inset shows the corresponding FFT pattern, indicating the [111] orientation. **e** Energy-dispersive X-ray spectroscopy (EDS) elemental mapping images of ultrathin Mn_x_Fe_y_Co_3-x-y_O_4_ alloy. Scale bars: **c**, **d** 0.5 nm; **e** 1 nm.

TTCG model can also be applied to multi-element oxides. Multi-element oxides crystallize into similar spinel-type structures, where metal ions occupy the centers of tetrahedrons or octahedrons sites surrounded by oxygen atoms (Fig. 4a). Taking CoFe_2_O_4_ as an example, the adsorption of water significantly influences ${\varepsilon }_{c}$ (0.29 eV/Å^2^ as shown in Supplementary Table 4), and it is predicted to have a great potential to form 2D nanoflakes with a negative ∆*E* of −0.53 eV/Å^2^ along the [111] direction (Fig. 4b). The X-ray diffraction (XRD) pattern of CoFe_2_O_4_ (Supplementary Fig. 7) can be indexed to the cubic structure with lattice parameters of a = b = c = 8.39 Å, and nanoflakes are well aligned with the [111] direction, in accord with the prediction by TTCG model.

Figure 4c–e shows the STEM-ADF and energy-dispersive X-ray spectroscopy (EDS) elemental mapping images of ultrathin CoFe_2_O_4_ and Mn_x_Fe_y_Co_3-x-y_O_4_ alloy, respectively. The STEM images and FFT patterns exhibit perfect hexagonally arranged lattice fringes and high-quality single-crystalline phases with the [111] orientation (Fig. 4c, d), the same as the predicted orientation by the model (Fig. 4b). Due to the complicated atoms distribution of spinel-type structure and the similar atomic number of Co/Fe/Mn, the actual positions of different elements are difficult to be discriminated. In addition, the nanoscale EDS elemental mappings confirm that Mn, Fe, Co, and O are uniformly distributed throughout the entire crystal (Fig. 4e and Supplementary Fig. 7c). Cross-section STEM (Supplementary Fig. 8) results show that the surface of CoFe_2_O_4_ is likely to be passivated by the hydroxyl group, which further demonstrates the significant role of ${\varepsilon }_{c}$ (by water) for promoting 2D growth and is in line with the model as well.

The successful growth of multi-element oxides illustrates that TTCG model is not limited by chemical composition and can be applied to guide multi-element nonlayered materials growth. The detailed characterizations of other oxides and oxyhalides or chalcogens are provided in Supplementary Figs. 9–22.

### Magnetic properties of oxides

**Fig. 5:**
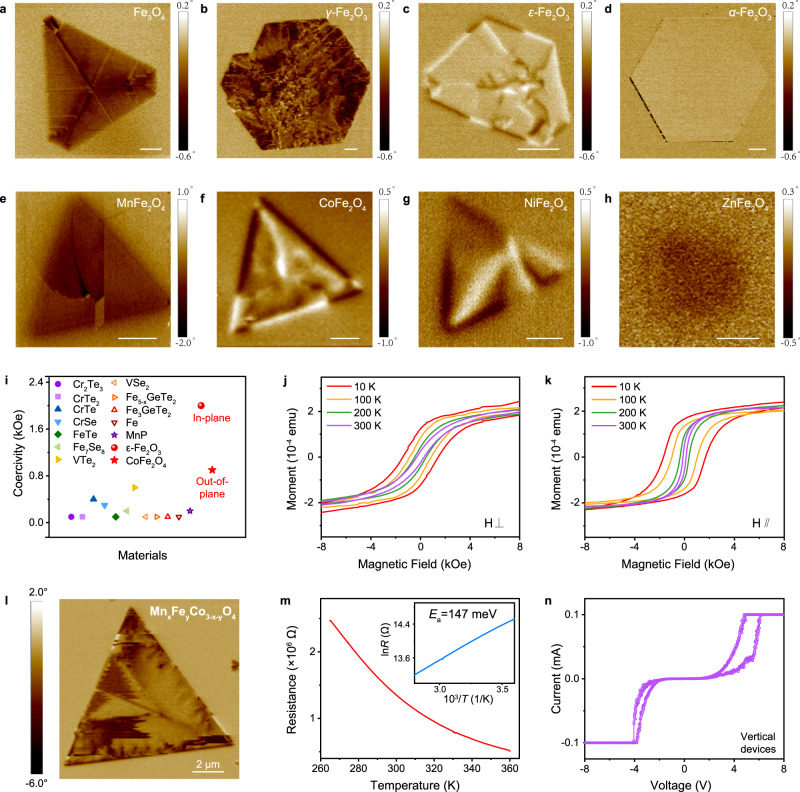
Room-temperature magnetism and electrical properties of oxides. **a**–**h** Magnetic force microscope (MFM) phase images of Fe_3_O_4_ (7.8 nm), *γ*-Fe_2_O_3_ (7.3 nm), *ε*-Fe_2_O_3_ (10 nm), *α*-Fe_2_O_3_ (27 nm), MnFe_2_O_4_ (20 nm), CoFe_2_O_4_ (18 nm), NiFe_2_O_4_ (15 nm), ZnFe_2_O_4_ (29 nm), respectively. Scale bars: **a**–**d**, 2 μm; **e**–**h**, 1 μm. **i** Comparison of coercivity at 200 K in *ε*-Fe_2_O_3_ and CoFe_2_O_4_ with other 2D magnets. **j** Magnetic hysteresis of Mn_x_Fe_y_Co_3-x-y_O_4_ alloy with magnetic field perpendicular to the substrate. **k** Magnetic hysteresis of Mn_x_Fe_y_Co_3-x-y_O_4_ alloy with magnetic field parallel to the substrate. **l** Room-temperature MFM phase image of Mn_x_Fe_y_Co_3-x-y_O_4_ alloy (16.2 nm). **m** Temperature-dependent longitudinal resistance of Mn_x_Fe_y_Co_3-x-y_O_4_ alloy from 260 K to 360 K. Inset: Fitted curves by Arrhenius equation, indicating the activation energy of 147 meV. **n** Current–voltage characteristic sweep of the vertical device under ambient conditions. The current compliance is 0.1 mA.

The successful synthesis of various TMO nanoflakes under the guidance of TTCG model has also enabled us to explore their unique properties. 2D TMO nanoflakes possess rich magnetic properties, ranging from antiferromagnets to soft magnets and hard magnets, which are characterized in Supplementary Figs. 23–25 and summarized in Supplementary Table 8. Importantly, most of them exhibit fascinating room-temperature magnetic ordering with high stability. In principle, oxides are more stable against air and water corrosion than chalcogens and chlorides. As exhibited in Supplementary Fig. 26, the surface topography of iron oxides shows no obvious oxidation after exposure to air for three months. To further demonstrate the room-temperature magnetism of 2D oxides nanoflakes, we measured their magnetic domain structure via MFM. As indicated in Fig. 5a–d and Supplementary Figs. 27–29, four iron oxides exhibit distinct domain patterns, confirming the realization of phase-controllable growth by our HACVD method from another perspective: Fe_3_O_4_ possesses out-of-plane triangular magnetic domains, and *γ*-Fe_2_O_3_ shows out-of-plane dendritic-like magnetic domains. Differently, flux-closure magnetic domains are imaged in *ε*-Fe_2_O_3_. The MFM phase contrast between *ε*-Fe_2_O_3_ and substrates is weaker, and the magnetic signal across the line profile exhibits double peaks (Supplementary Fig. 28), indicating the in-plane magnetism of *ε*-Fe_2_O_3_. However, it is hard to discern the magnetic signals of *α*-Fe_2_O_3_, attributed to its antiferromagnetic behavior^19^. Moreover, the observation of magnetic domain signals in ultrathin ferrites (Fig. 5e–h) also provides evidence for the existence of room-temperature magnetism in ternary oxides nanoflakes. Different compositions have disparate magnetic signal intensity and domain states.

It is worth noting that *ε*-Fe_2_O_3_ and CoFe_2_O_4_ are both hard magnets, the coercivity of which is larger than most reported 2D magnets^5,39–49^, as shown in Fig. 5i and Supplementary Fig. 30. The discovered high-temperature 2D magnets are rare, let alone one owning large coercivity above 200 K, which has great application prospects in high-density magnetic recording and storage. As for *ε*-Fe_2_O_3_, the coercivity is ~2000 Oe in the in-plane direction. Moreover, out-of-plane coercivity of CoFe_2_O_4_ is determined to be ~900 Oe. The thickness-dependent magnetic properties of CoFe_2_O_4_ are investigated by reflection magnetic circular dichroism (RMCD) and MFM measurements in Supplementary Fig. 31. With reducing the thickness, the magnetism becomes weaker, the coercivity decreases, and multi-domain structure gets more sophisticated.

In addition, alloying can further extend the magnetic material family. As depicted in Fig. 5j, k, obvious hysteresis was observed in Mn_x_Fe_y_Co_3-x-y_O_4_ nanoflakes regardless of applying a perpendicular or parallel magnetic field, showing the room-temperature magnetic ordering and large coercivity. Temperature-dependent magnetization curves illustrate the ferrimagnetic behavior of Mn_x_Fe_y_Co_3-x-y_O_4_ alloy (Supplementary Fig. 32). The multi-domain magnetic states in MFM image further demonstrate the room-temperature magnetism of an individual Mn_x_Fe_y_Co_3-x-y_O_4_ nanoflake, which is well maintained with thickness even down to 3.5 nm (Fig. 5l and Supplementary Fig. 33). To explore the electrical properties, devices are fabricated on Mn_x_Fe_y_Co_3-x-y_O_4_ nanoflakes transferred to SiO_2_/Si substrate (Supplementary Fig. 34). The anomalous Hall effect and butterfly-shaped hysteresis behavior of magneto-resistance curves (Supplementary Fig. 35) also indicate the appearance of magnetic ordering in Mn_x_Fe_y_Co_3-x-y_O_4_. Figure 5m displays the temperature-dependent resistance ($R$) which increases gradually with decreasing temperatures, declaring the typical semiconductor feature. By fitting the Arrhenius equation (${{{{{\rm{ln}}}}}}{R}={{{{\mathrm{ln}}}}}{R}_{0}+{E}_{a}/{k}_{B}T$, where ${k}_{B}$ is Boltzmann constant, *T* is the temperature, and ${R}_{0}$ is the fitting parameter), the activation energy (${E}_{a}$) is estimated to be 147 meV. Resistive switching behavior is also observed in the vertical devices of Mn_x_Fe_y_Co_3-x-y_O_4_ alloy (Fig. 5n and Supplementary Fig. 36). When the voltage sweeps back and forth, the current does not overlap, illustrating the semiconductor behavior as well.

## Discussion

In summary, we have proposed a TTCG model to get a new insight into the synthesis of 2D materials. It offers a multivariate quantitative criterion, which can not only predict the growth modes but also provide powerful guidance for experiments. For instance, specific crystal orientation, the adoption of water vapor, and the selection of substrate are considered to promote the 2D anisotropic growth of oxides. In addition, this model is not limited by crystal structures or element compositions, which is generalized both to layered and especially to nonlayered materials (including oxides, chalcogens, and oxyhalides), promoting the theoretical research and chemical synthesis of 2D materials.

Guided by the model, we design a universal HACVD strategy to synthesize a family of 2D nonlayered TMO nanoflakes, including 9 binary, 4 ternary, and 6 alloy oxides. Moreover, phase-controllable growth of Fe-based oxides (Fe_3_O_4_ and *γ*-, *ε*-, *α*-Fe_2_O_3_) can also be achieved. Importantly, the attracting room-temperature magnetic ordering, high stability, rich magnetic domain structure, larger coercivity, and semiconductivity of as-synthesized oxides nanoflakes can further expand the family of 2D magnets, and provide alternative platforms for pioneering applications in room-temperature magnetoelectronic or spintronic devices.

## Methods

### Synthesis of oxides

2D iron oxides were synthesized on mica substrates via hydrate-assisted CVD method. The reaction was conducted in a one-inch quartz tube heated by a three-zone furnace (Lindberg/Blue M). ~800 mg CaSO_4_·2H_2_O powder (99.5%, Alfa Aesar) was put at the upstream as the water source, the temperature of which is set at ~130 °C. ~15 mg Ferrous chloride (99.5%, Alfa Aesar) was placed in an alumina boat downstream. Freshly cleaved fluorophlogopite mica (Taiyuan Fluorphlogopite Mica Company Ltd, 10 × 10 × 0.2 mm) is used as the substrate and put 1–3 cm away from ferrous chloride. Prior to the growth, the furnace was purged by 400 sccm high-purity Ar gas for 15 min to remove the residual air. Then, 100 sccm Ar were inlet as the carrier gas during the whole growth process. O_2_ was introduced into the CVD system at the beginning of heating for different times to synthesize different phases of iron oxides. The growth zone of ferrous chloride was heated to 550–700 °C for 20 min, and kept for another 10 min for growth. As for other oxides, corresponding metal chlorides are employed, and the growth conditions are similar with iron oxides. The detailed synthesis recipes of transition-metal-based oxides are provided in Supplementary Table 9.

### Synthesis of oxyhalide

50 mg EuCl_3_·6H_2_O or SmCl_3_·6H_2_O (98%, Alfa Aesar) and 20 mg NaCl were mixed together in an alumina boat where the temperature is set at 900 °C. Mica was placed just over the precursors with the growth time of 20 min. 20 sccm Ar was used as the carrier gas to grow EuOCl or SmOCl.

### Synthesis of chalcogens

20 mg CrCl_3_ (98%, Alfa Aesar) and 50 mg Te (99.9%, Alfa Aesar) powders were mixed together to synthesize Cr_5_Te_8_. Precursors were placed in a quartz boat at 700 °C with a mica substrate placed face-down. Before heating, the CVD system was evacuated and filled with Ar three times to remove oxygen and moisture. Then, mixed gases of 5 sccm H_2_ and 100 sccm Ar were introduced for growth. Once the reaction ended, the furnace was moved away for rapid cooling. Besides, FeCl_2_ (Alfa Aesar, 99.5%) and S (Alfa Aesar, 99.5%) powder were used as the Fe and S source to synthesize Fe_7_S_8_. 100 mg S powder was placed in the first heating zone at 130 °C, and 20 mg FeCl_2_ was placed in an alumina boat in the third heating zone at 560 °C. Mica was put 1 cm downstream away from FeCl_2_. Before heating, the CVD system was evacuated and filled with Ar three times to remove oxygen and moisture. 10 sccm H_2_ and 100 sccm Ar were introduced and the heating time is set at ~10 min.

### Characterizations

Optical images were characterized by Nexcope NM910 microscope. Atomic force microscope (Bruker, Dimension Icon) was employed to measure thicknesses, and MFM is conducted by the magnetic force modes with magnetic tips. EDS mapping (FEI, Tecnai F30) was used to analyze element compositions. Raman spectra were collected by Horiba, XploRA PLUS with excitation light of ~532 nm. Magnetism was characterized by the physical property measurement system (DynaCool, Quantum Design) equipped with vibrating sample magnetometry.

The atomic structures of ferrites were characterized by a cold-field emission transmission electron microscope (JEOL ARM200F) operating at 200 kV. The convergent semi-angle for the incident probe was set about 30 mrad. The collection angle of the ADF images ranges from 81 to 228 mrad.

### Device fabrication and electrical transport measurement

Oxides nanoflakes were transferred from mica to Si/SiO_2_ substrates via poly(methyl methacrylate)-assisted method. Then, devices were defined by standard e-beam lithography (FEI NanoSEM). 5 nm Ti and 60 nm Au were deposited by e-beam evaporation to be adopted as the contact electrodes. For vertical devices, 20 nm Au was used for bottom electrodes. Two-electrode testings were carried out on a probe station (Lakeshore TTP4) equipped with a vacuum pump and Keithley 4200 semiconductor analyzer. Magneto-transport measurements were conducted by four-probe electrical measurements in a commercial physical property measurement system (DynaCool, Quantum Design) with magnetic field vertical to the sample and temperature from 260 K to 360 K.

### Density functional theory calculations

The geometrical optimizations were performed by DFT based on Perdew-Burke-Ernzerhof generalized gradient approximation (PBE-GGA) functional^50^, implemented in Vienna Ab-initio Simulation Package (VASP)^51^. The electronic properties were described by the projector augmented wave (PAW) potentials with a kinetic energy cutoff of 500 eV for planewave basis set, as confirmed by a convergence test^52^. The valence electron configurations for Cr (3*p*^6^3*d*^5^4*s*^1^), Fe (3*p*^6^3*d*^6^4*s*^2^), Co (3*d*^7^4*s*^2^), Zn (3*d*^10^4*s*^2^), Eu (4*f* ^7^5*s*^2^5*p*^6^6*s*^2^), Sm (4*f* ^6^5*p*^6^6*s*^2^), O (2*s*^2^2*p*^4^), S (3*s*^2^3*p*^4^), Te (5*s*^2^5*p*^4^), Cl (3*s*^2^3*p*^5^), and H (1*s*^1^) were employed. The first Brillouin zone is represented by a Γ-point-centered Monkhorst–Pack ***k****-*mesh^53^ with a grid density of $2{\pi} \times 0.02$ Å^−1^ along each dimension. The strong correlation in the 3d orbitals of Fe, Co, Cr atoms are treated by including an on-site Coulomb interaction of $U=4.0,3.3,3.5$ eV^54^, respectively. The non-local van der Waals (vdW) interactions were corrected with the Grimme’s zero damping DFT-D3 method^55^. The dipole-dipole interaction corrections in the *z* axis were incorporated^56^. The tolerance of 1.0 × 10^−5^ eV for self-consistent-field (SCF) iteration was set, and the atoms of subunit slabs were allowed to be fully relaxed until all the force components decreased below 0.02 eV/Å, without any symmetry constraints.

## Supplementary information

Suporting Information

## Data Availability

Relevant data supporting the key findings of this study are available within the article, the Supplementary Information file, and the Source data file. All raw data generated during the current study are available from the corresponding authors upon request. Source data are provided with this paper.

## Source data

Source data
