# Supplementary material for: A general thermodynamics-triggered competitive growth model to guide the synthesis of two-dimensional nonlayered materials
Source: Nat Commun. 2023 Feb 21;14:958. doi: 10.1038/s41467-023-36619-5 (PMC9944324; doi:10.1038/s41467-023-36619-5)
Supplement: Supplementary file 1 — Suporting Information [file 41467_2023_36619_MOESM1_ESM.pdf]

Supplementary Information

A general thermodynamics-triggered competitive growth model to guide the synthesis of two-dimensional nonlayered materials

Zijing Zhao[1,2,#], Zhi Fang[1,#], Xiaocang Han[1,#], Shiqi Yang[3], Cong Zhou[4], Yi Zeng[1], Biao Zhang[1], Wei Li[1], Zhan Wang[5], Ying Zhang[5], Jian Zhou[4], Jiadong Zhou[6], Yu Ye[3], Xinmei Hou[7], Xiaoxu Zhao[1*], Song Gao[8], Yanglong Hou[1,2*]

[1]School of Materials Science and Engineering, Beijing Key Laboratory for Magnetoelectric Materials and Devices, Beijing Innovation Center for Engineering Science and Advanced Technology, Peking University, Beijing 100871, China.

[2]Academy for Advanced Interdisciplinary Studies, Peking University, Beijing 100871, China.

[3]State Key Laboratory for Mesoscopic Physics and Frontiers Science Center for Nano-Optoelectronics, School of Physics, Peking University, Beijing 100871, China.

[4]Center for Alloy Innovation and Design, State Key Laboratory for Mechanical Behavior of Materials, Xi'an Jiaotong University, Xi'an 710049, China.

[5]Beijing National Laboratory for Condensed Matter Physics, Institute of Physics, Chinese Academy of Sciences, Beijing 100190, China.

[6]Centre for Quantum Physics, Key Laboratory of Advanced Optoelectronic Quantum Architecture and Measurement, School of Physics, Beijing Institute of Technology, Beijing 100081, China.

[7]Innovation Research Institute for Carbon Neutrality, University of Science and Technology Beijing, Beijing 100083, China.

[8]Institute of Spin-X Science and Technology, South China University of Technology, Guangzhou 510641, China.

*Corresponding authors, e-mails: xiaoxuzhao@pku.edu.cn; hou@pku.edu.cn

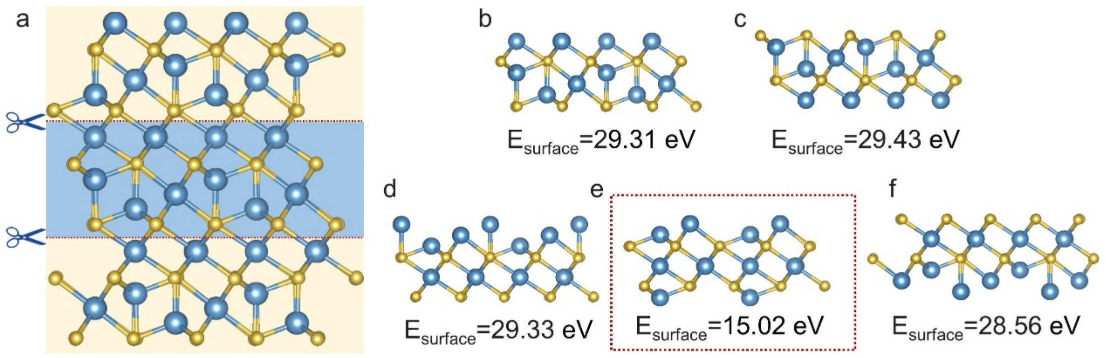

Supplementary Fig. 1. Different subunit configurations of Fe_3O_4 in the [111] direction. a, The Fe_3O_4 (111) slab with one subunit highlighted by blue. **b-f**, Different subunits with various terminals and their corresponding surface energies. Blue and yellow spheres represent Fe and O atoms, respectively.

The average surface energies of different subunits with various terminals are calculated by the following equation:

$$E_{\mathrm{surface}}=E_{\mathrm{slab}}-N*E_{\mathrm{unit}}/2$$

where E_{surface}, E_{slab}, E_{unit} and N are the surface energy, the energy of slab, the energy of unit cell, and the number of unit cells in the slab, respectively.

The selection of subunit is more complex and needs to be discussed individually in nonlayered materials. Different crystal orientations may have different subunits. At the same orientation, surface configurations also have an influence on the system energy[1]. Taking Fe_3O_4 as an example. As is shown in Supplementary Fig. 1, different subunits with various terminals in the [111] direction have distinct energies. And the Fe-terminated subunit with lowest surface energy (e) is considered to be the optima configuration, which is used in the following model.

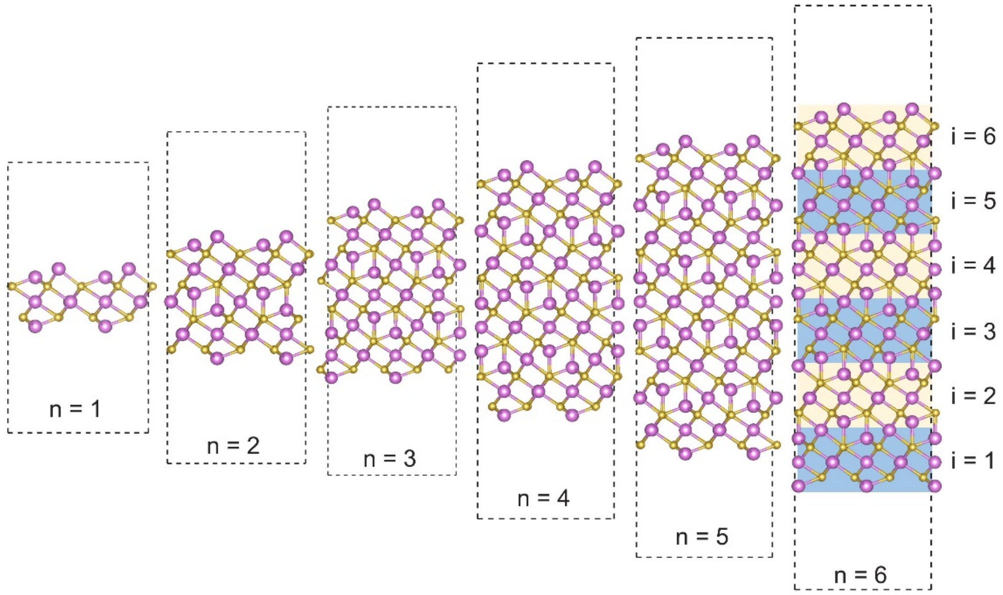

Supplementary Fig. 2. The configurations of Fe_3O_4 with *n* subunits (*n*=1-6) in the [111] direction. Each subunit is labeled as *i* (*i* = 1-*n*) from bottom to top in *n* subunits stacked Fe_3O_4 slab. Pink and yellow spheres represent Fe and O atoms, respectively.

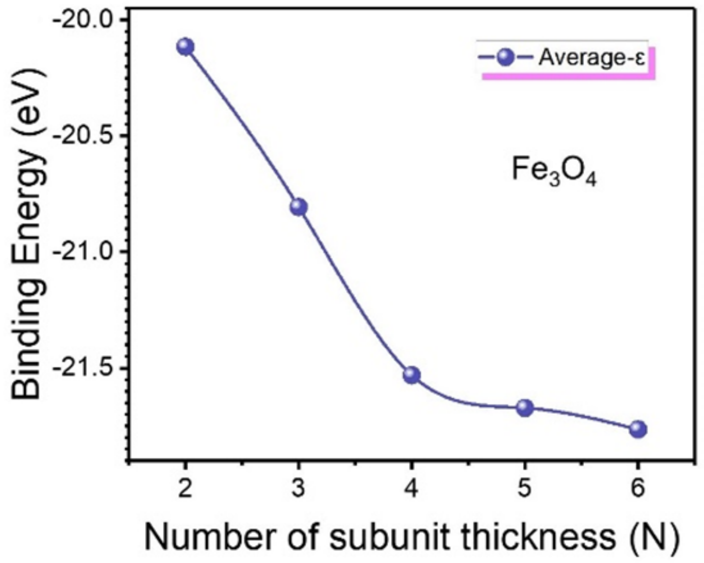

Supplementary Fig. 3. The relationship between $\varepsilon_{i,i+1}$ and *n* in Fe_3O_4. $\varepsilon_{i,i+1} = E_{i+1} - E_i$ (E_i is the total energy of subunit with the thickness of *n* in Supplementary Fig. 2).

The covalent binding in nonlayered materials is stronger, so the interaction force can act to next units and $\varepsilon_{i,i+1}$ is theoretically related with *n*. As calculated in Supplementary Fig. 3, the value gets dramatically enhanced with the raise of *n* when $n \leq 4$, while it hardly changes as *n* further increases ($n > 4$).

The interaction force of $\varepsilon_{i,i+1}$ is in the vertical direction, thus the increase of vertical thickness (*i.e.*, *n*) will inevitably enhance $\varepsilon_{i,i+1}$. By contrast, λ_i is determined by horizontal interaction force, which is mainly affected by the diameter of materials instead of the thickness, so the effect of *n* on λ_i can be ignored.

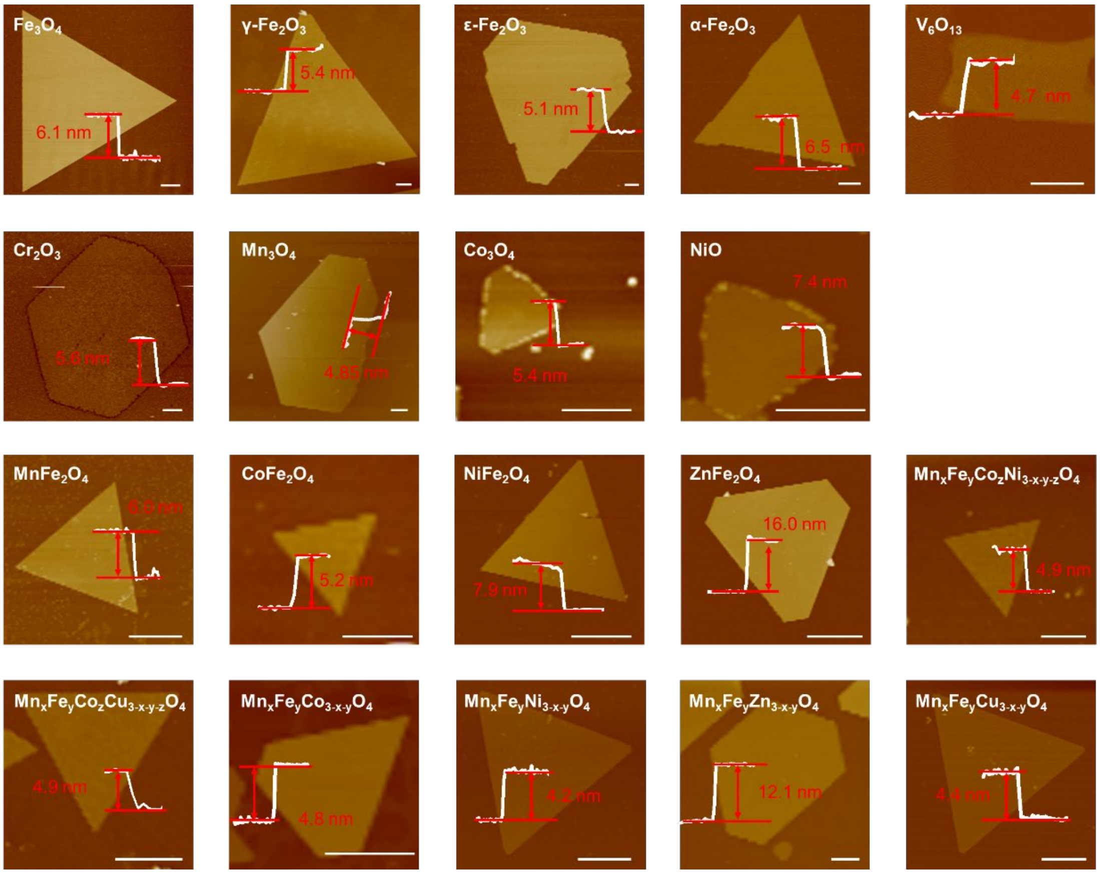

Supplementary Fig. 4. AFM topological images of as-synthesized 2D oxides in Fig. 2b. The scale bars are 1 μm.

Most 2D oxides can be down to 4-7 nanometers (~3-5 unit cells). Zn-based multi-element oxides are relatively thicker, because $ZnCl_2$ precursor has a lower melting point incompatible with other chlorides, leading to more volatilization of precursors and larger thickness. Besides, all oxides are stable in the air with no obvious oxidation dots observed and the surface is smooth, indicating the high quality of our synthesized oxides.

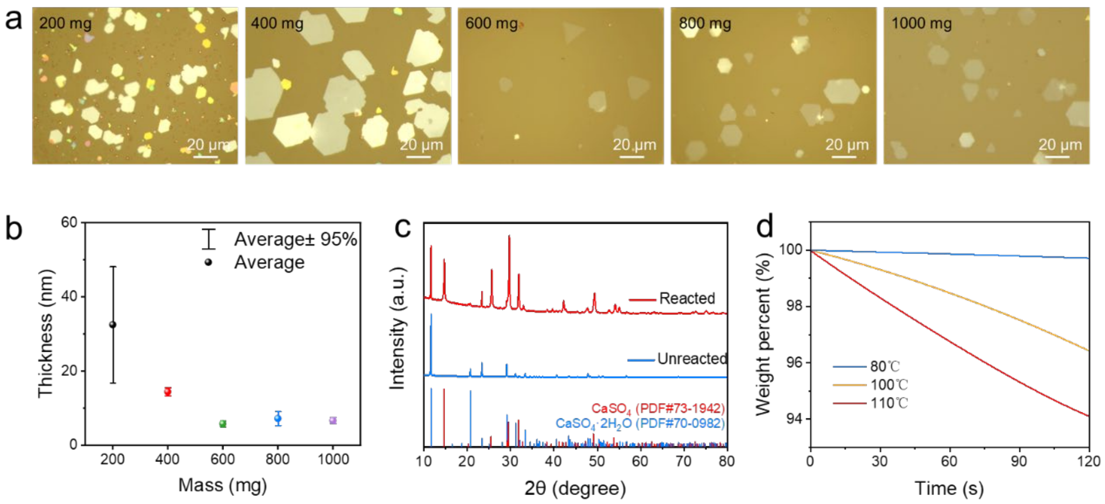

Supplementary Fig. 5. The thickness regulation of HACVD method. a, OM images of *ε*-Fe_2O_3 using different masses of $CaSO_4{\cdot}2H_2O$. **b**, The thickness regulation with the change of hydrate mass. **c**, XRD pattern of $CaSO_4{\cdot}2H_2O$ before and after reaction (the original mass is ~800 mg). **d**, Thermogravimetric curve of $CaSO_4{\cdot}2H_2O$ at different temperatures.

Taking *ε*-Fe_2O_3 as an example here (Supplementary Fig. 5a, b), the thickness can be largely reduced with increasing the mass of hydrates, indicating the important role of H_2O as well. When the hydrates mass reaches 800 mg, the change of mass makes less effect.

After the reaction, XRD peaks of anhydrous compound appear in addition to unreacted hydrates (Supplementary Fig. 5c), illustrating that water is released upon heating $CaSO_4{\cdot}2H_2O$ and is adequate for the reaction when the mass exceeds 800 mg. Therefore, we speculate the following mechanism: On the one hand, hydrates release water vapor to react with chlorides in the gaseous atmosphere to form oxides on substrates. On the other hand, sufficient water molecules adsorb on the surface of oxides and decrease *ε* term (Fig. 1d-h), thus 2D growth is preferred and the thickness is reduced. Moreover, we found that $CaSO_4{\cdot}2H_2O$ can release water at different rates by varying the temperatures (Supplementary Fig. 5d), served as a stable source of water vapor in a much controller rate range and lower vapor pressure than liquid water without the usage of complicated setups.

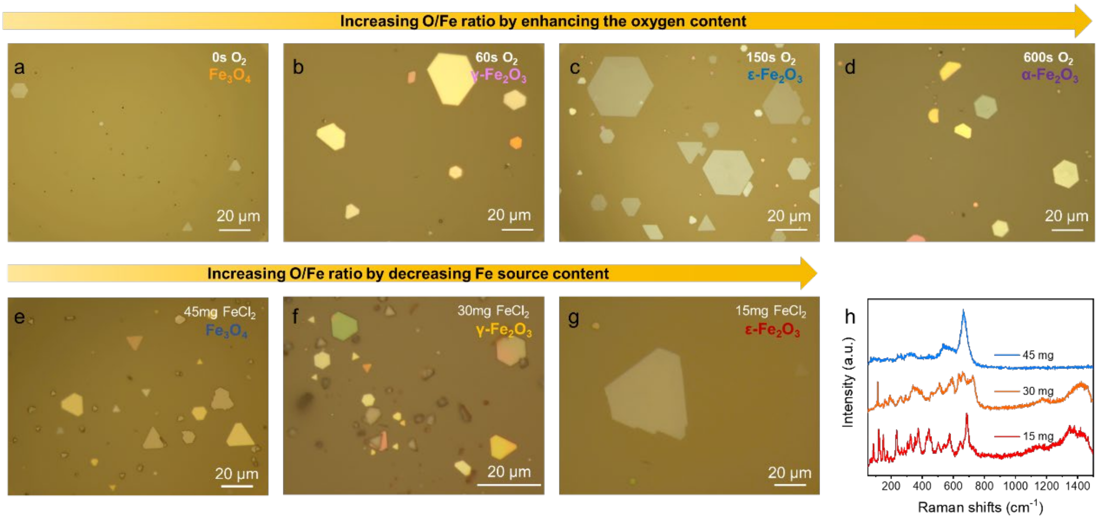

Supplementary Fig. 6. Phase regulation of different Fe-based oxides. a-d, OM images of iron oxides by enhancing the duration of oxygen at the same mass of $FeCl_2$ (15 mg). **e-g**, OM images under different masses of Fe source at the same oxygen concentration (The oxygen intake time is 120 s). **h**, Corresponding Raman spectra of the samples on (e-g).

With the increment of oxygen content (O/Fe ratio is increased), 2D Fe_3O_4, γ-Fe_2O_3, ε-Fe_2O_3, and α-Fe_2O_3 nanoflakes can be grown, respectively (Supplementary Fig. 6a-d). The corresponding Raman spectra of the samples on Supplementary Fig. 6a-d are shown in Fig. 3b-e. Moreover, with decreasing the mass of $FeCl_2$ at the same oxygen concentration, the obtained phases transfer from Fe_3O_4 to γ-Fe_2O_3 and further to ε-Fe_2O_3. α-Fe_2O_3 is obtained at higher oxygen concentrations (The oxygen intake time is 600 s). These results demonstrate that regulating the O/Fe ratio can realize phase-controllable synthesis of iron oxides.

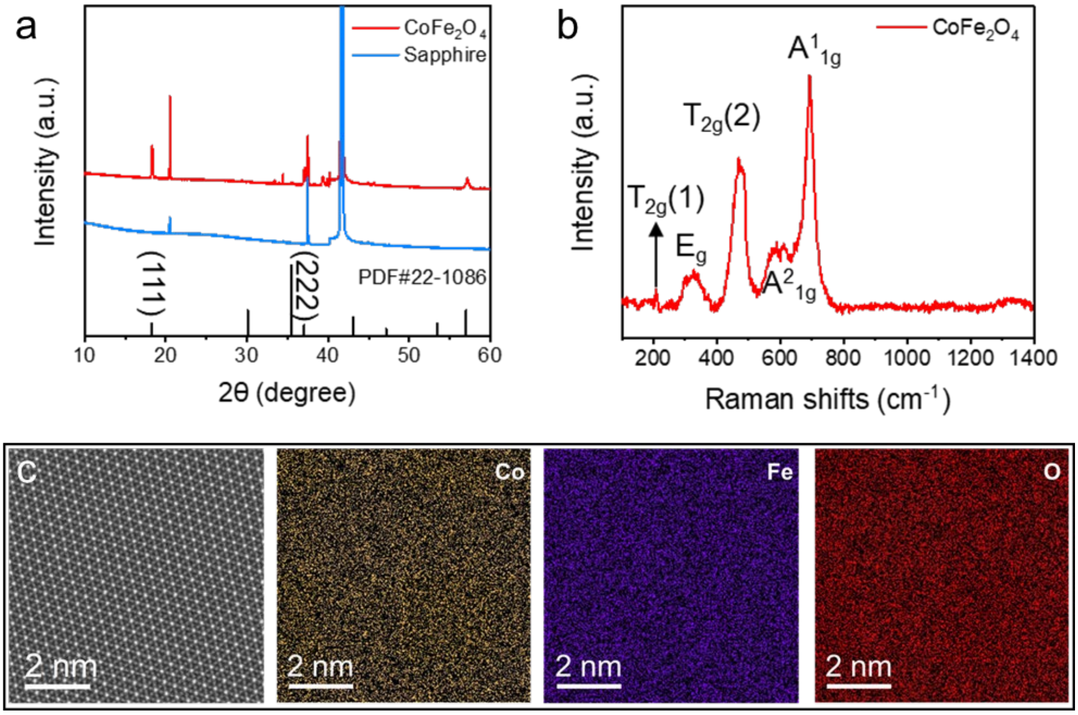

Supplementary Fig. 7. Structural characterizations of 2D $CoFe_2O_4$ nanoflakes. a, XRD pattern of as-synthesized nanoflakes on sapphire substrates. The two primary diffraction peaks are indexed to the (111) and (222) planes, illustrating that nanoflakes are well aligned with the [111] direction. **b**, Raman spectrum of $CoFe_2O_4$ nanoflake. The vibration modes are labeled in the figure. **c**, STEM and corresponding nanoscale EDS elemental mapping images of ultrathin $CoFe_2O_4$ along the [111] direction.

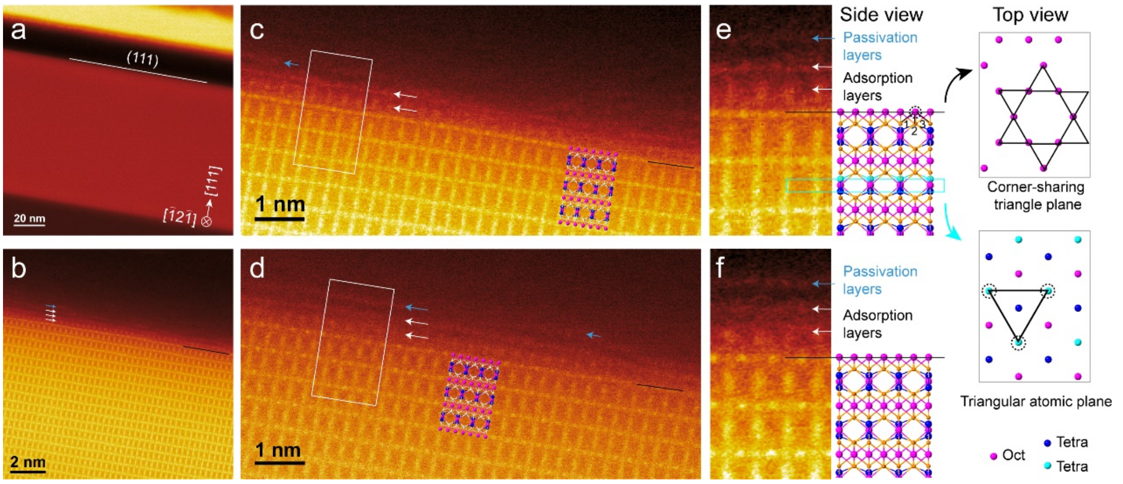

Supplementary Fig. 8. Cross-sectional STEM of $CoFe_2O_4$ nanoflakes. a, Low magnification STEM images of the cross-sectional $CoFe_2O_4$. **b-d**, Atomic scaled (111) surface structure from different areas in (a) viewed along [$\bar{1}2\bar{1}$] direction, inserted with the atomic model. **e, f**, Enlarged atomic structure of (111) surface from the white box region of (c, d). The right panels of (e, f) display the side view of (111) lattice planes. From the top view, we can see that the octahedral cation sublattice consists of alternating triangular and corner-sharing triangular atomic planes. The orange, pink, and blue (cyan as well) spheres represent O atoms, octahedral sites, and tetrahedral sites, respectively.

The cross-section sample was prepared by Focused Ion Beam (FIB) milling, and the sectioning line is almost perfectly parallel to one side of the triangular sample. As shown in Supplementary Fig. 8a, the side view is along [$\bar{1}2\bar{1}$] direction, revealing the (111) surface structure of the nonlayered nanosheet. Based on the Z-contrast ADF-STEM image (Supplementary Fig. 8c, d), we find that the stable (111) surface planes are consistently terminated by octahedral Fe (or Co) cations (pink atoms) with corner-sharing triangles lattice (marked by the black lines). Interestingly, extra components (Fe, O, and Co) absorbed on the surface, which was confirmed by the relatively weak contrast along the outmost surface plane (marked by white arrows on the top surface). In other words, the top surface would possess growth steps (*i.e.* the incomplete growth layer) with the coverage less than 30% from normalized ADF image contrast. The number of adsorbed layers (the incomplete growth layer) varied from two to four based on the Z-contrast, and the atomic structure is similar to that of the bulk structure. In fact,

the adsorbed layers can spread over hundreds of nanometers, much larger than the height (0.48~1.0 nm), indicating the lateral growth of the nonlayered 2D materials as well.

Moreover, an additional layer (denoted by blue arrows) was also observed on the topmost surface, and the arrangement is disordered compared to the below growth layers, which is likely to be the passivation layer of hydroxyl group, because there were large amounts of water vapor during the CVD synthesis process. Thus, the dangling bonds on the surface are decreased (less than three) as a result of passivation, where the saturated coordination number for octahedral sites is six and three O atoms have already coordinated with Fe (or Co) beneath the surface (Supplementary Fig. 8e, f). Due to the weak contrast, the actual number of adsorbed O elements on the octahedral Fe (or Co) surface is hard to distinguish.

In a word, the top surface of oxides has growth steps terminated by octahedral metal (Fe or Co) cations. The dangling bonds on the surface are decreased, and the surface atoms are speculated to be passivated by hydroxyl group from the STEM images.

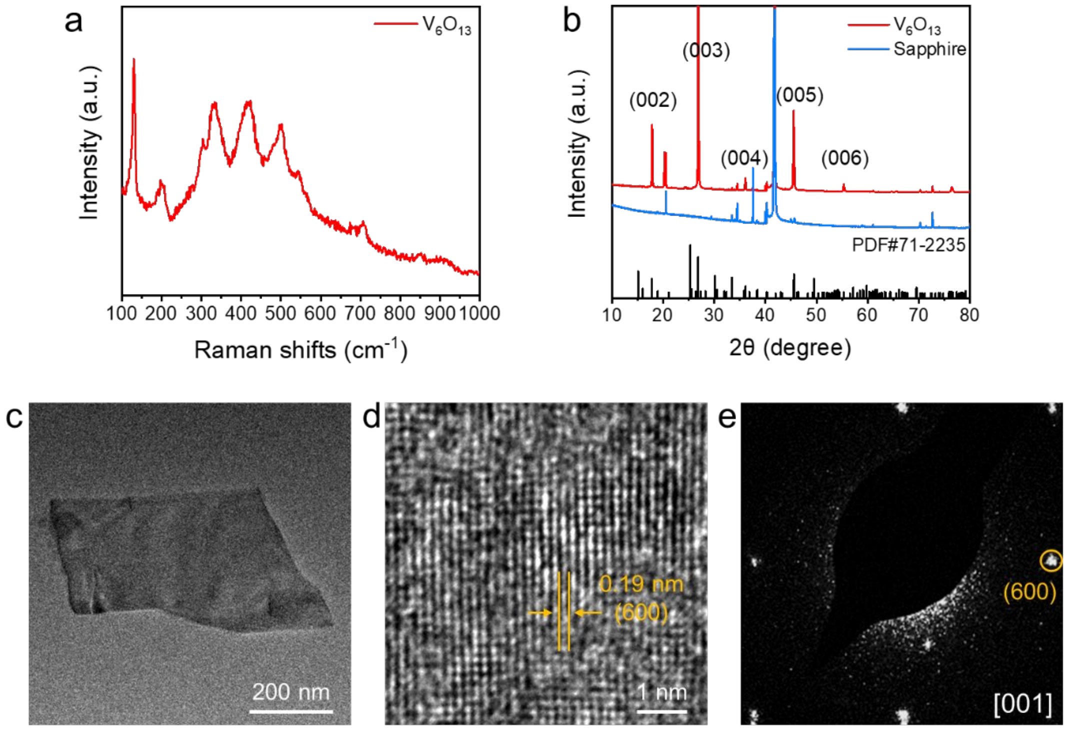

Supplementary Fig. 9. Structural characterizations of 2D V_6O_{13} nanoflakes. a, Raman spectrum of the nanoflake, which demonstrates the formation of V_6O_{13}[2]. **b**, XRD pattern of as-synthesized nanoflakes on sapphire substrates. The major diffraction peaks are indexed to the (00l) planes, illustrating that nanoflakes are well aligned with the [001] direction. **c**, Typical transmission electron microscope (TEM) image of V_6O_{13} nanoflake. **d**, High-resolution transmission electron microscope (HRTEM) image showing the lattice spacing of 0.19 nm, attributed to the (600) crystal plane. **e**, Corresponding SAED pattern indicating the [001] orientation.

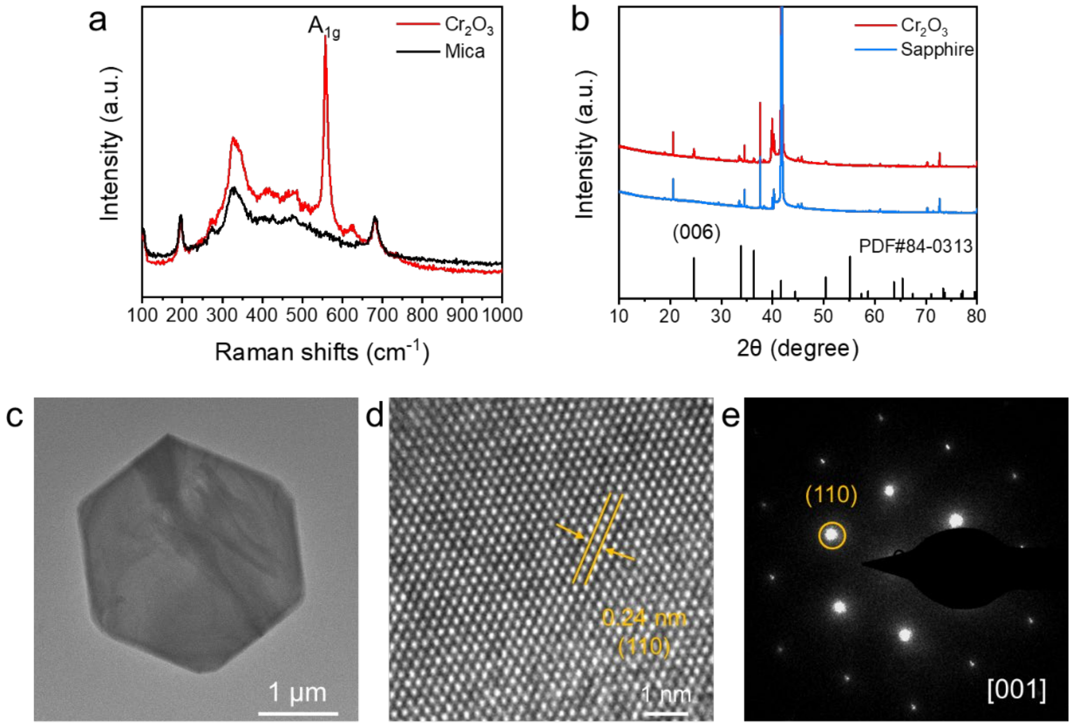

Supplementary Fig. 10. Structural characterizations of 2D Cr_2O_3 nanoflakes. a, Raman spectrum of Cr_2O_3 nanoflake. The A_{1g} vibration mode is labeled in the figure. **b**, XRD pattern of as-synthesized nanoflakes on sapphire substrates. The primary diffraction peak corresponds to the (006) planes of Cr_2O_3. **c**, Typical TEM image of Cr_2O_3 nanoflake. **d**, HRTEM image showing the lattice spacing of 0.24 nm, attributed to the (110) crystal plane. **e**, Corresponding SAED pattern indicating the [001] orientation.

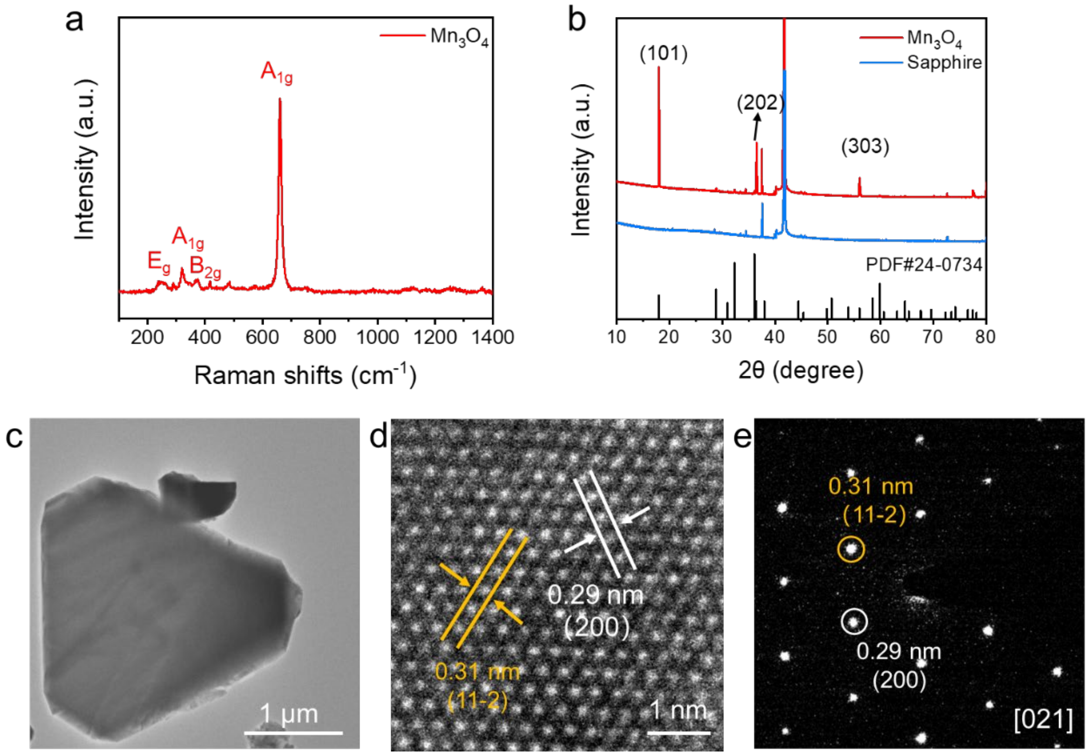

Supplementary Fig. 11. Structural characterizations of 2D Mn_3O_4 nanoflakes. a, Raman spectrum of Mn_3O_4 nanoflake. The vibration modes are labeled in the figure. **b**, XRD pattern of as-synthesized nanoflakes on sapphire substrates, demonstrating the formation of Mn_3O_4. **c**, Typical TEM image of Mn_3O_4 nanoflake. **d**, HRTEM image showing the lattice spacing of 0.29 nm and 0.31 nm, attributed to the (200) plane and (11-2) plane. **e**, Corresponding SAED pattern indicating the [021] orientation.

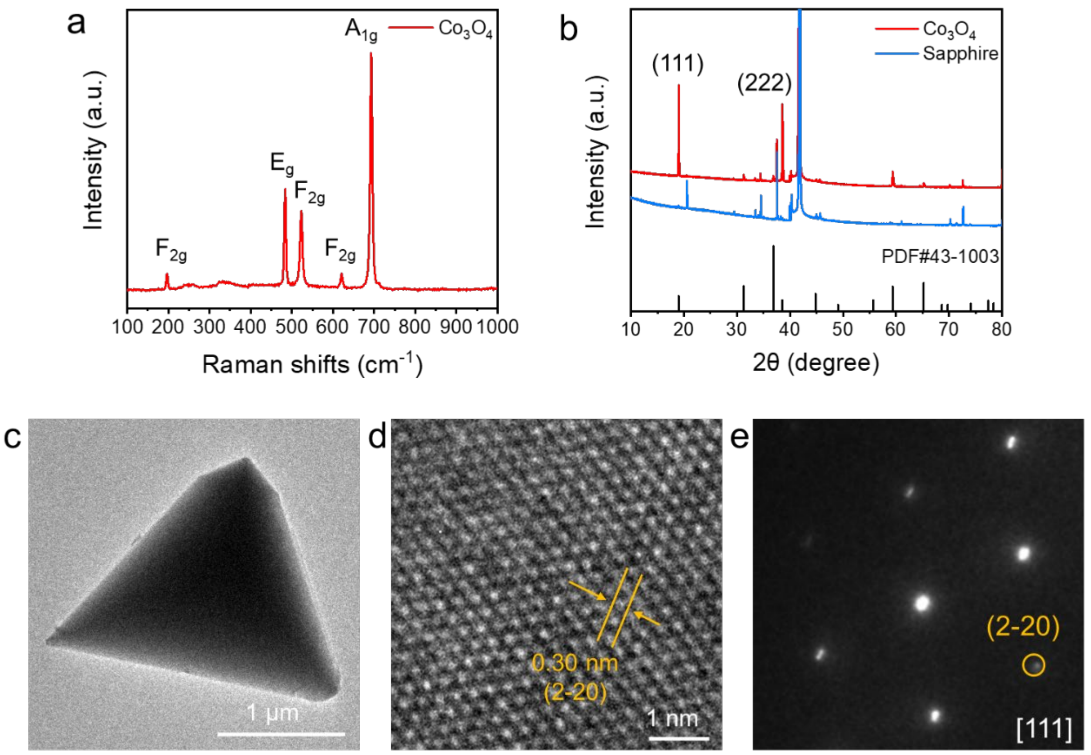

Supplementary Fig. 12. Structural characterizations of 2D Co_3O_4 nanoflakes. a, Raman spectrum of Co_3O_4 nanoflake. The vibration modes are labeled in the figure. **b**, XRD pattern of as-synthesized nanoflakes on sapphire substrates. The two primary diffraction peaks are indexed to the (111) and (222) planes, illustrating that nanoflakes are well aligned with the [111] direction. **c**, Typical TEM image of Co_3O_4 nanoflake. **d**, HRTEM image showing the lattice spacing of 0.30 nm, attributed to the (2-20) crystal plane. **e**, Corresponding SAED pattern indicating the [111] orientation.

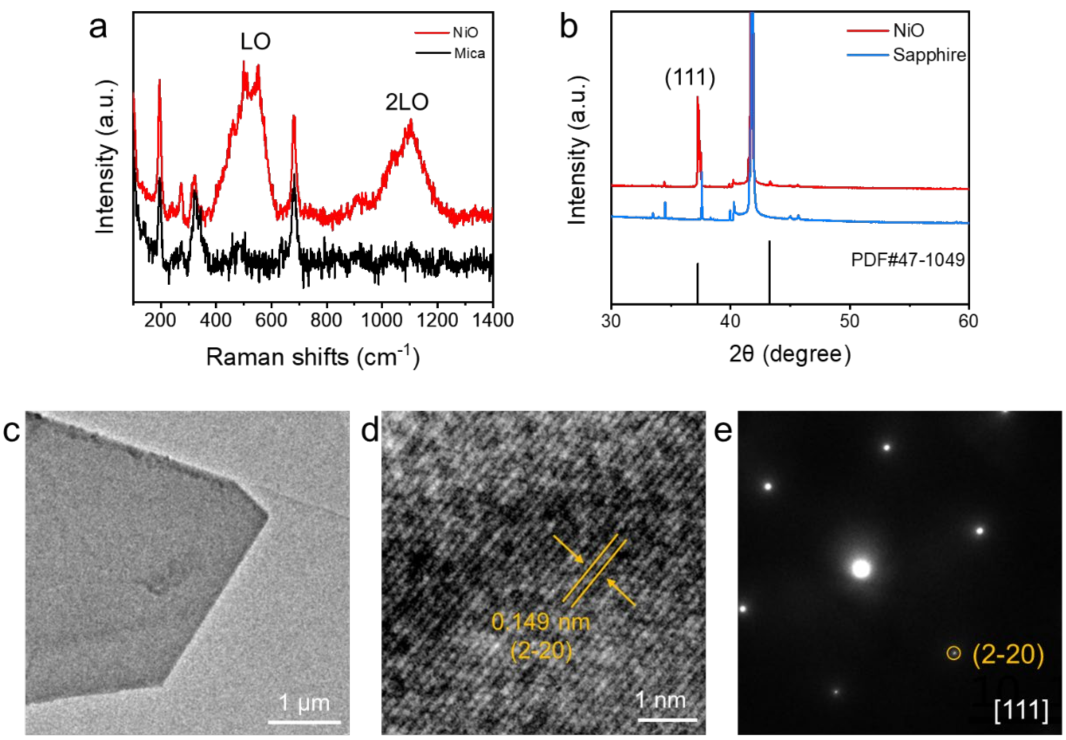

Supplementary Fig. 13. Structural characterizations of 2D NiO nanoflakes. a, Raman spectrum of NiO nanoflake. The vibration modes are labeled in the figure. **b**, XRD pattern of as-synthesized nanoflakes on sapphire substrates. The major diffraction peak is indexed to the (111) plane, illustrating that nanoflakes are well aligned with the [111] direction. **c**, Typical TEM image of NiO nanoflake. **d**, HRTEM image showing the lattice spacing of 0.149 nm, attributed to the (2-20) crystal plane. **e**, Corresponding SAED pattern indicating the [111] orientation.

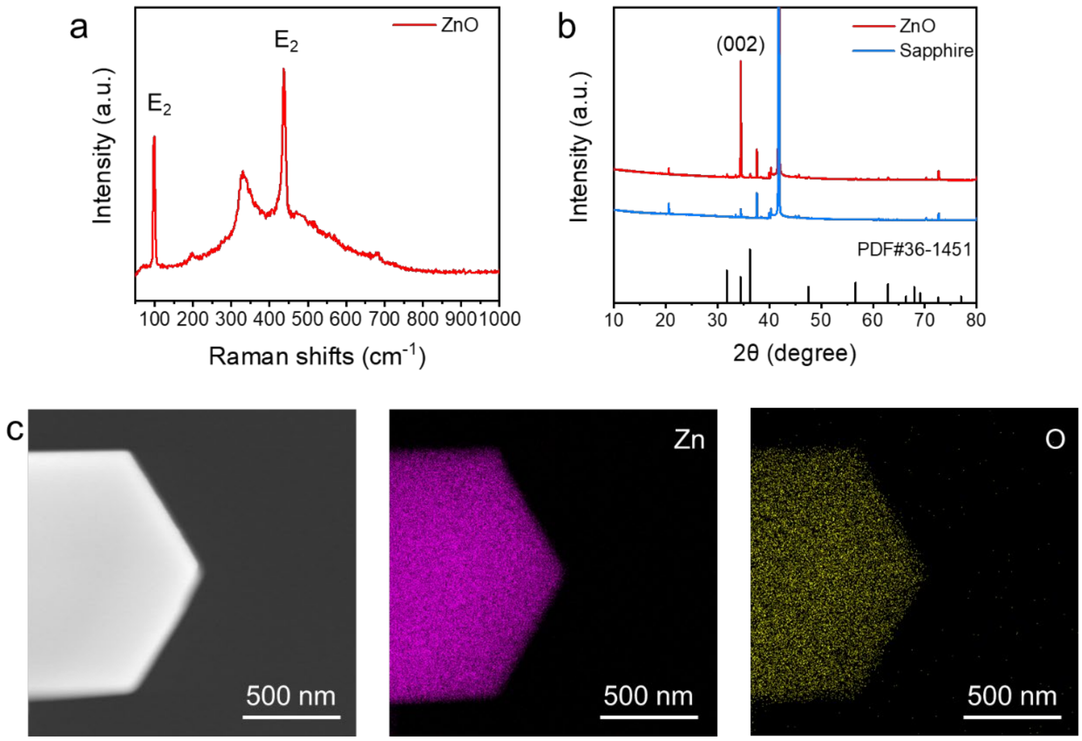

Supplementary Fig. 14. Structural characterizations of ZnO nanorods. a, Raman spectrum of ZnO nanorod. The vibration modes are labeled in the figure. **b**, XRD pattern of as-synthesized nanorods on sapphire substrates. The major diffraction peak is indexed to the (002) plane, illustrating that ZnO is well aligned with the [001] direction. **c**, HAADF image and corresponding EDS elemental mappings.

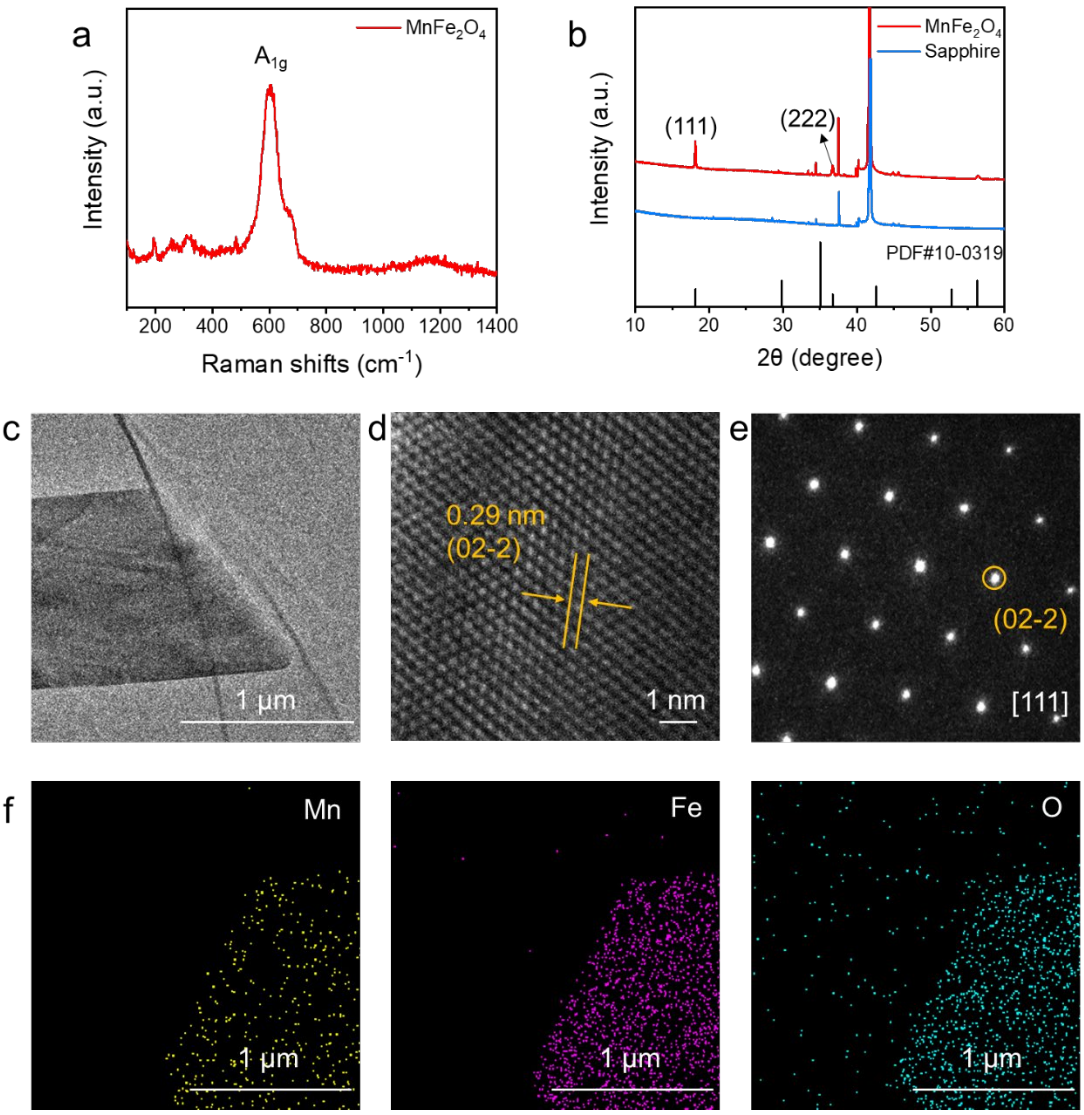

Supplementary Fig. 15. Structural characterizations of 2D $MnFe_2O_4$ nanoflakes. **a**, Raman spectrum of $MnFe_2O_4$ nanoflake. The vibration modes are labeled in the figure. **b**, XRD pattern of as-synthesized nanoflakes on sapphire substrates. The two primary diffraction peaks are indexed to the (111) and (222) planes, illustrating that nanoflakes are well aligned with the [111] direction. **c**, Typical TEM image of $MnFe_2O_4$ nanoflake. **d**, HRTEM image showing the lattice spacing of 0.29 nm, attributed to the (02-2) crystal plane. **e**, Corresponding SAED pattern indicating the [111] orientation. **f**, EDS elemental mappings of Mn, Fe, and O.

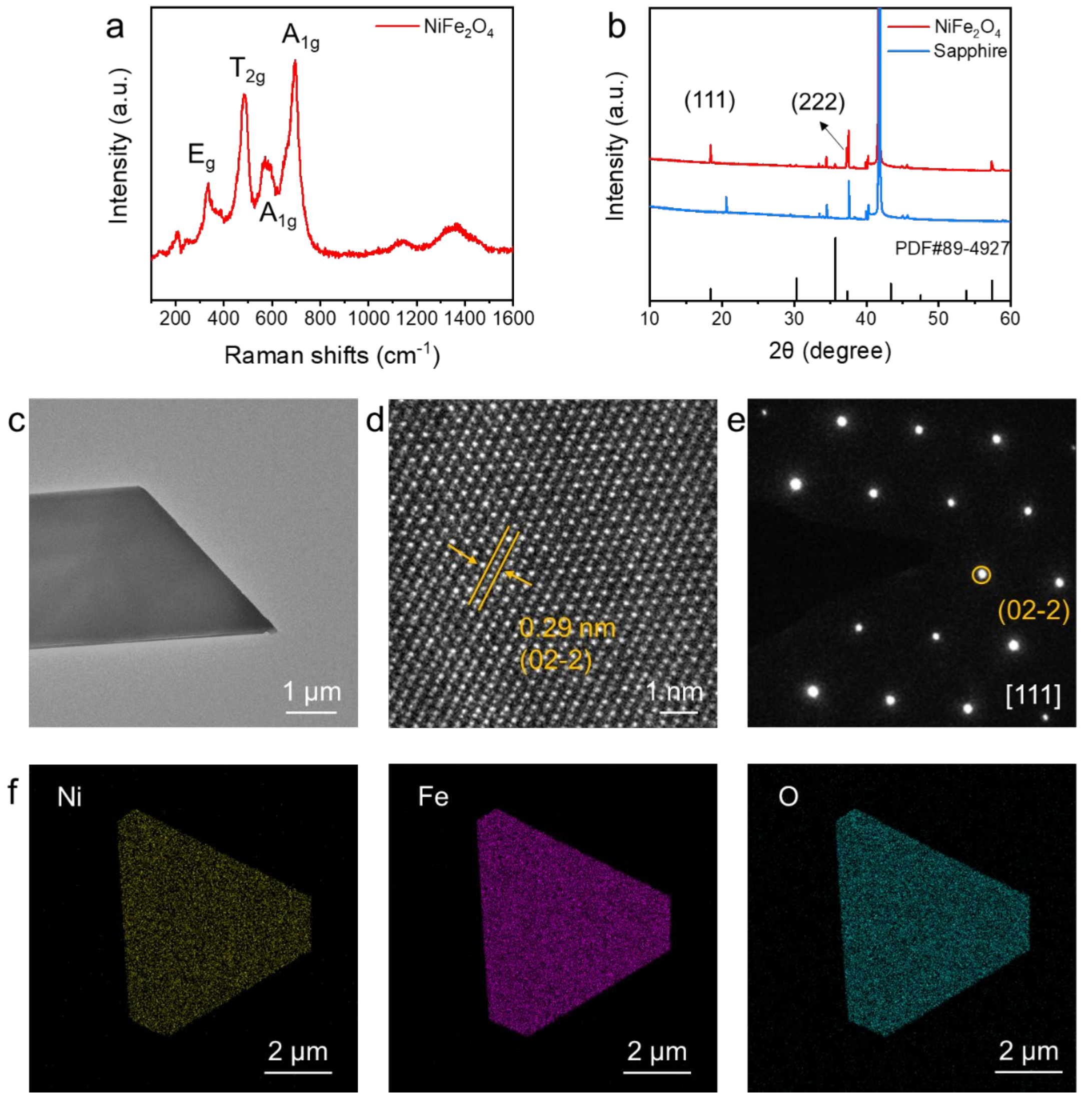

Supplementary Fig. 16. Structural characterizations of 2D $NiFe_2O_4$ nanoflakes. a, Raman spectrum of $NiFe_2O_4$ nanoflake. The vibration modes are labeled in the figure. **b**, XRD pattern of as-synthesized nanoflakes on sapphire substrates. The two primary diffraction peaks are indexed to the (111) and (222) planes, illustrating that nanoflakes are well aligned with the [111] direction. **c**, Typical TEM image of $NiFe_2O_4$ nanoflake. **d**, HRTEM image showing the lattice spacing of 0.29 nm, attributed to the (02-2) crystal plane. **e**, Corresponding SAED pattern indicating the [111] orientation. **f**, EDS elemental mappings of Ni, Fe, and O.

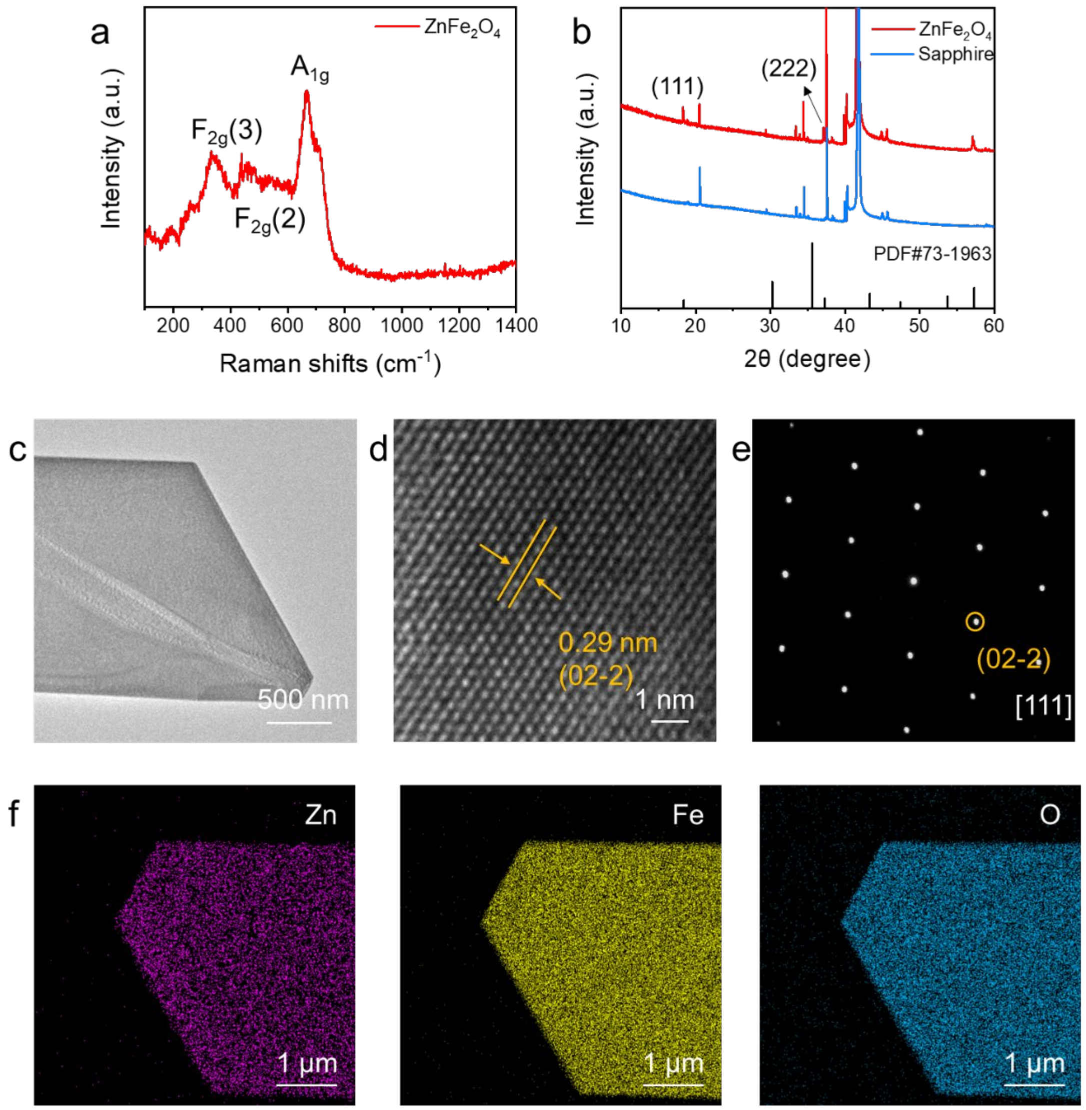

Supplementary Fig. 17. Structural characterizations of 2D $ZnFe_2O_4$ nanoflakes. a, Raman spectrum of $ZnFe_2O_4$ nanoflake. The vibration modes are labeled in the figure. **b**, XRD pattern of as-synthesized nanoflakes on sapphire substrates. The two primary diffraction peaks are indexed to the (111) and (222) planes, illustrating that nanoflakes are well aligned with the [111] direction. **c**, Typical TEM image of $ZnFe_2O_4$ nanoflake. **d**, HRTEM image showing the lattice spacing of 0.29 nm, attributed to the (02-2) crystal plane. **e**, Corresponding SAED pattern indicating the [111] orientation. **f**, EDS elemental mappings of Zn, Fe, and O.

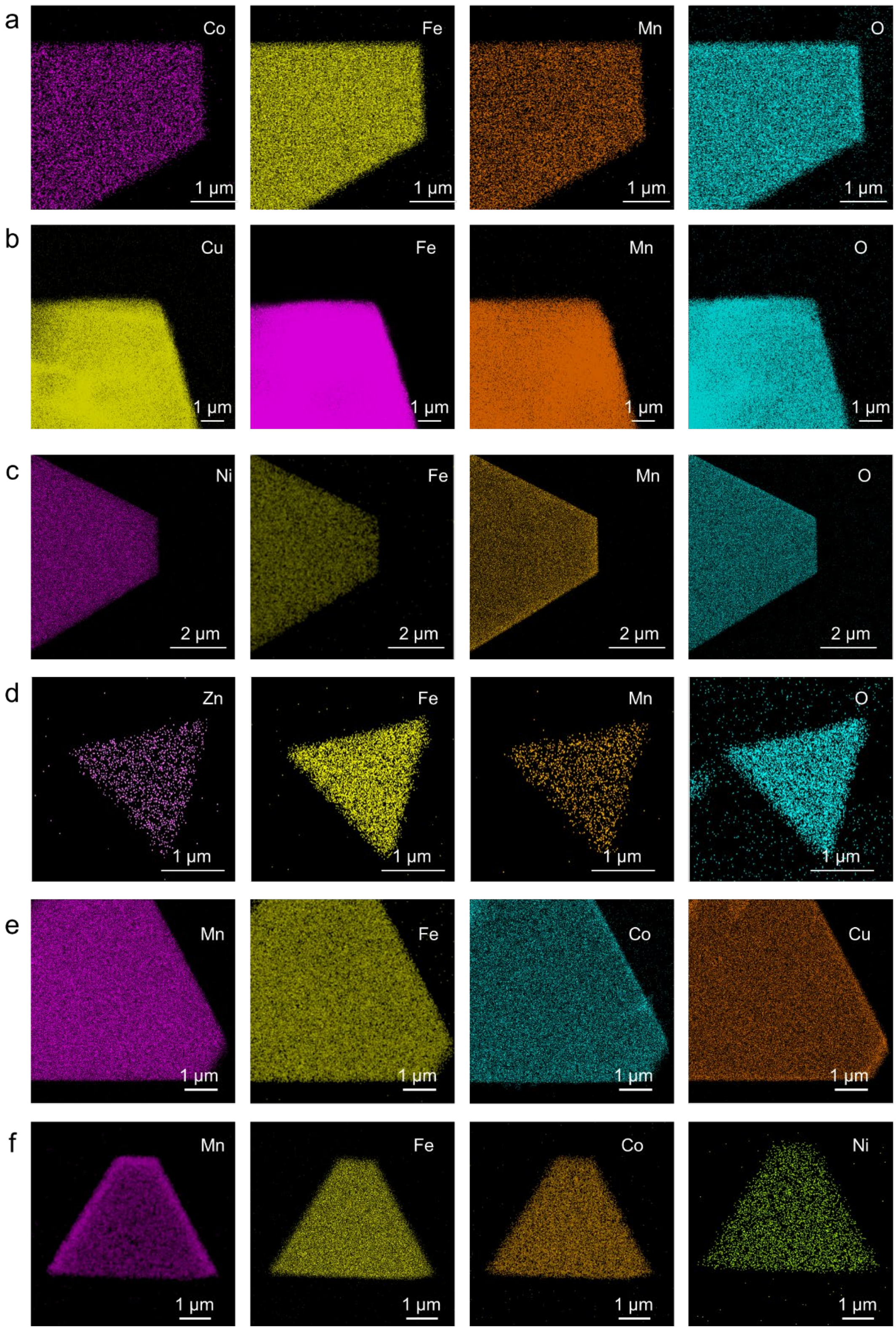

Supplementary Fig. 18. EDS elemental mappings of $Mn_xFe_yCo_{3-x-y}O_4$ alloy (a), $Mn_xFe_yCu_{3-x-y}O_4$ alloy (b), $Mn_xFe_yNi_{3-x-y}O_4$ alloy (c), $Mn_xFe_yZn_{3-x-y}O_4$ alloy (d), quinary MnFeCoCuO alloy (e), and MnFeCoNiO alloy (f), respectively.

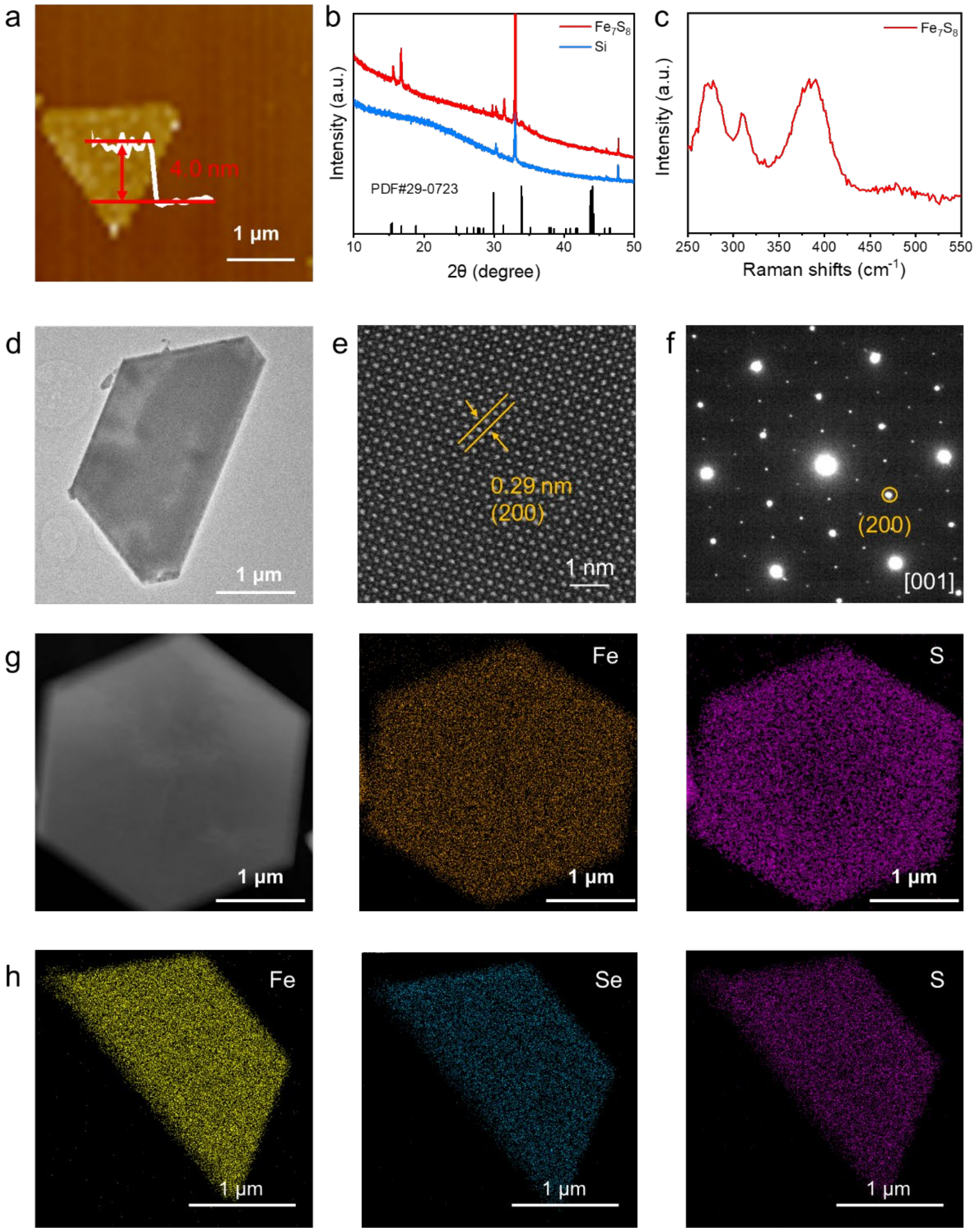

Supplementary Fig. 19. Structural characterizations of 2D Fe_7S_8 and FeSeS nanoflakes. a, AFM image of Fe_7S_8 nanoflake with the thickness of 4.0 nm. **b**, XRD pattern of as-synthesized nanoflakes transferred on Si substrates, demonstrating the synthesis of Fe_7S_8 phase. **c**, Typical Raman spectrum of Fe_7S_8. **d**, TEM image of Fe_7S_8 nanoflake. **e**, HRTEM image exhibiting a lattice spacing of 0.29 nm, attributed to the (200) crystal plane. **f**, Corresponding SAED pattern indicating the [001] orientation. The inner superspots illustrate that Fe:S is 7:8[3]. **g**, HAADF image and corresponding EDS elemental mappings of Fe and S. **h**, EDS elemental mappings of FeSeS alloy, indicating the uniform distribution of Fe, S, and Se elements.

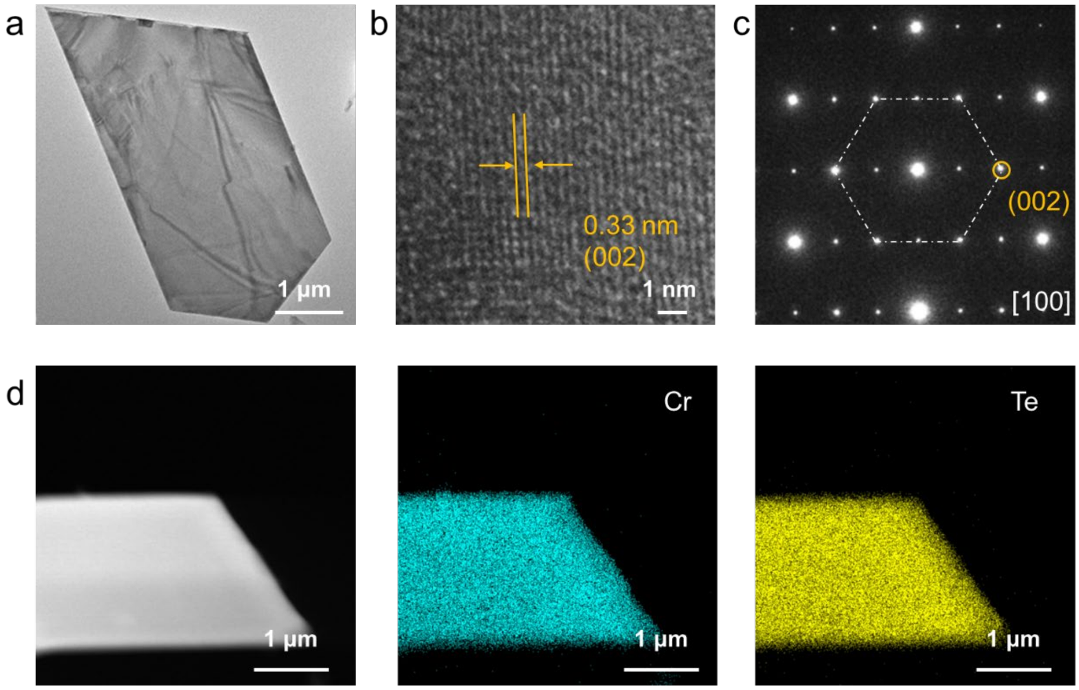

Supplementary Fig. 20. Structural characterizations of Cr_5Te_8 nanoflakes. a, TEM image of Cr_5Te_8 nanoflake. **b**, HRTEM image exhibiting a lattice spacing of 0.33 nm, attributed to the (002) crystal plane. **c**, Corresponding SAED pattern indicating the monoclinic phase structure and the [100] orientation[4]. **d**, HAADF image and corresponding EDS elemental mappings of Cr and Te.

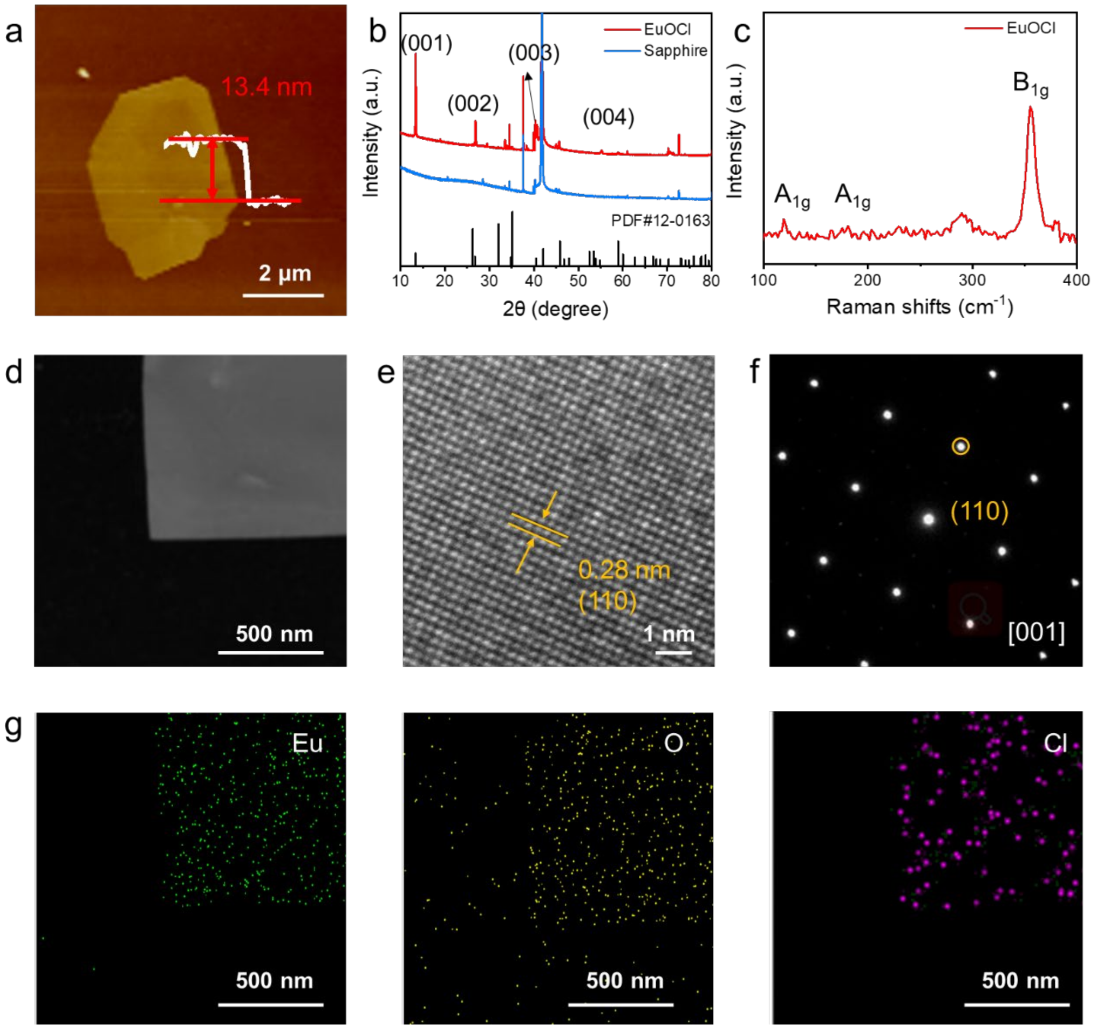

Supplementary Fig. 21. Structural characterizations of 2D EuOCl nanoflakes. a, AFM image of EuOCl with the thickness of 13.4 nm. **b**, XRD pattern of as-synthesized nanoflakes on sapphire substrates. The primary diffraction peaks are indexed to the (00l) planes, illustrating that nanoflakes are well aligned with the [001] direction. **c**, Typical Raman spectrum of EuOCl nanoflake. The vibration modes are labeled in the figure. **d**, TEM image of a quadrangle EuOCl nanoflake. **e**, HRTEM image exhibiting a lattice spacing of 0.28 nm, attributed to the (110) crystal plane. **f**, Corresponding SAED pattern indicating the [001] orientation. **g**, EDS elemental mappings of Eu, O, and Cl.

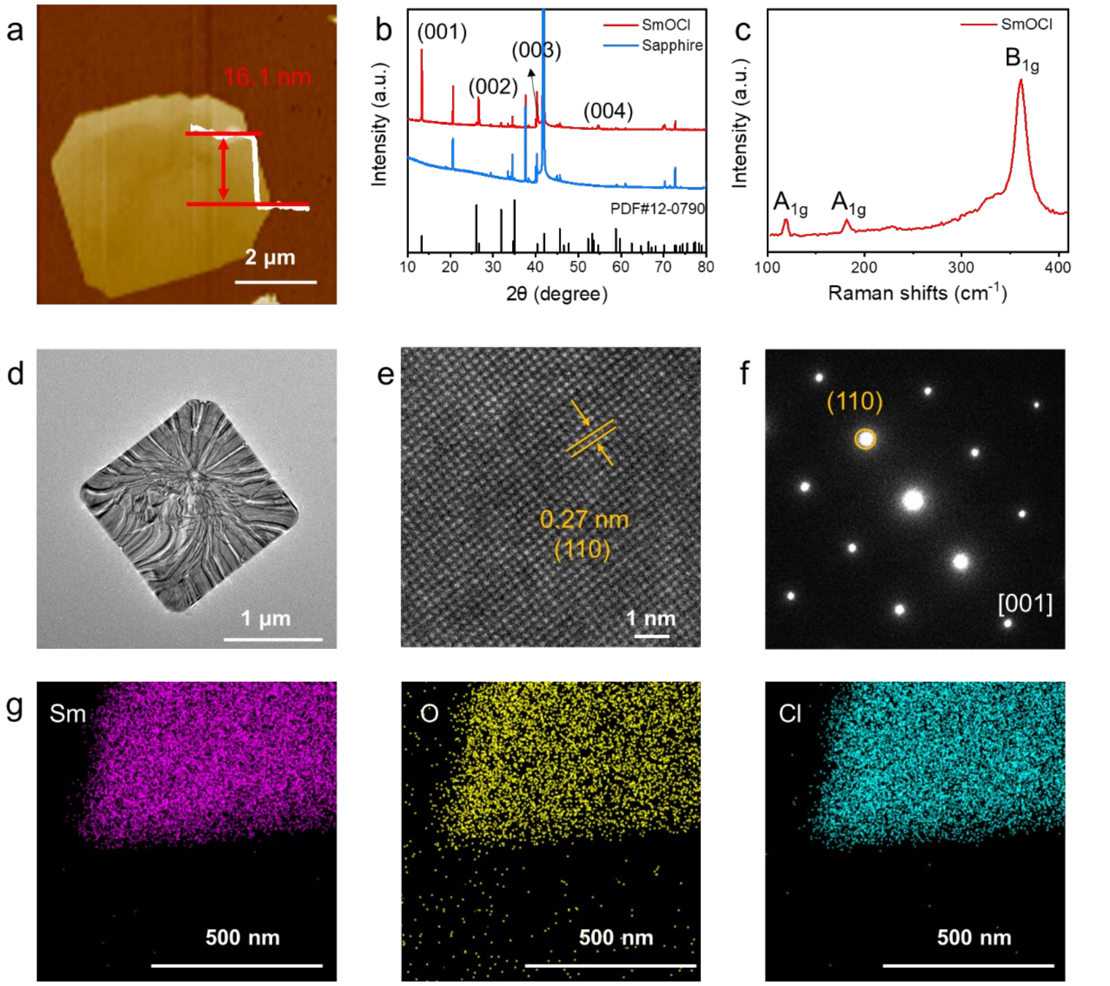

Supplementary Fig. 22. Structural characterizations of 2D SmOCl nanoflakes. **a**, AFM image of SmOCl with the thickness of 16.1 nm. **b**, XRD pattern of as-synthesized nanoflakes on sapphire substrates. The primary diffraction peaks are indexed to the (00l) planes, illustrating that nanoflakes are well aligned with the [001] direction. **c**, Typical Raman spectrum of SmOCl nanoflake. The vibration modes are labeled in the figure. **d**, TEM image of a quadrangle SmOCl nanoflake. **e**, HRTEM image exhibiting a lattice spacing of 0.27 nm, attributed to the (110) crystal plane. **f**, Corresponding SAED pattern indicating the [001] orientation. **g**, EDS elemental mappings of Sm, O, and Cl.

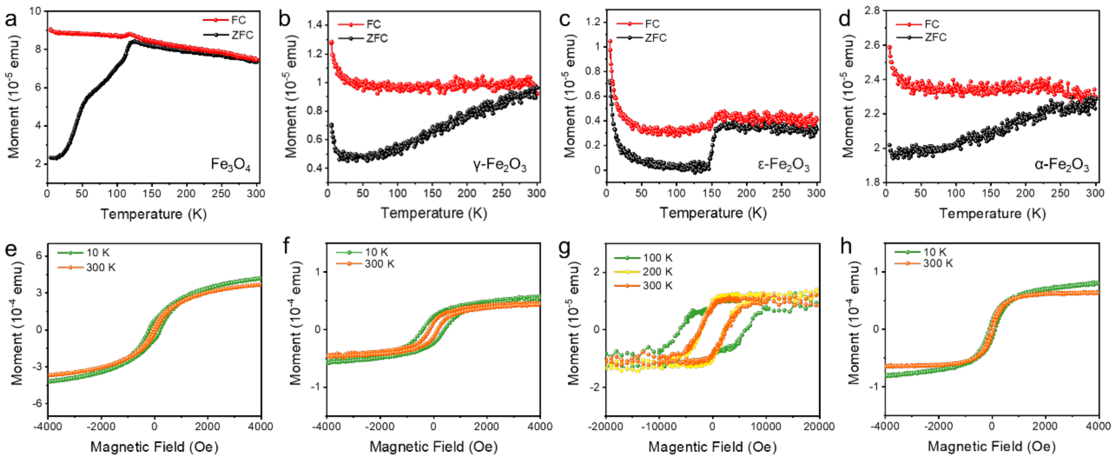

Supplementary Fig. 23. Magnetization curves of Fe-based oxides. a-d, Zero-field-cooled (ZFC) and field-cooled (FC) magnetization curves of Fe_3O_4 (a), γ-Fe_2O_3 (b), ε-Fe_2O_3 (c), and α-Fe_2O_3 (d), respectively, with the magnetic field of 200 Oe parallel to the substrate. **e-h**, Magnetization versus magnetic field curves at different temperatures of Fe_3O_4 (e), γ-Fe_2O_3 (f), ε-Fe_2O_3 (g), and α-Fe_2O_3 (h), respectively, after subtracting background signals at high fields.

Fe_3O_4, γ-Fe_2O_3, and ε-Fe_2O_3 all possess room-temperature ferrimagnetism because of the obvious magnetic hysteresis at 300 K. Besides, Fe_3O_4 has a sharp drop in ZFC and FC curves at ~120 K, known as Verwey transition[5]. γ-Fe_2O_3 has a small coercivity. ε-Fe_2O_3 is reported to be a collinear ferrimagnet with a metamagnetic transition at ~150 K[6]. In addition, α-Fe_2O_3 is antiferromagnetic coupling with weak magnetic ordering due to the uncompensated surface spin of 2D structure[7].

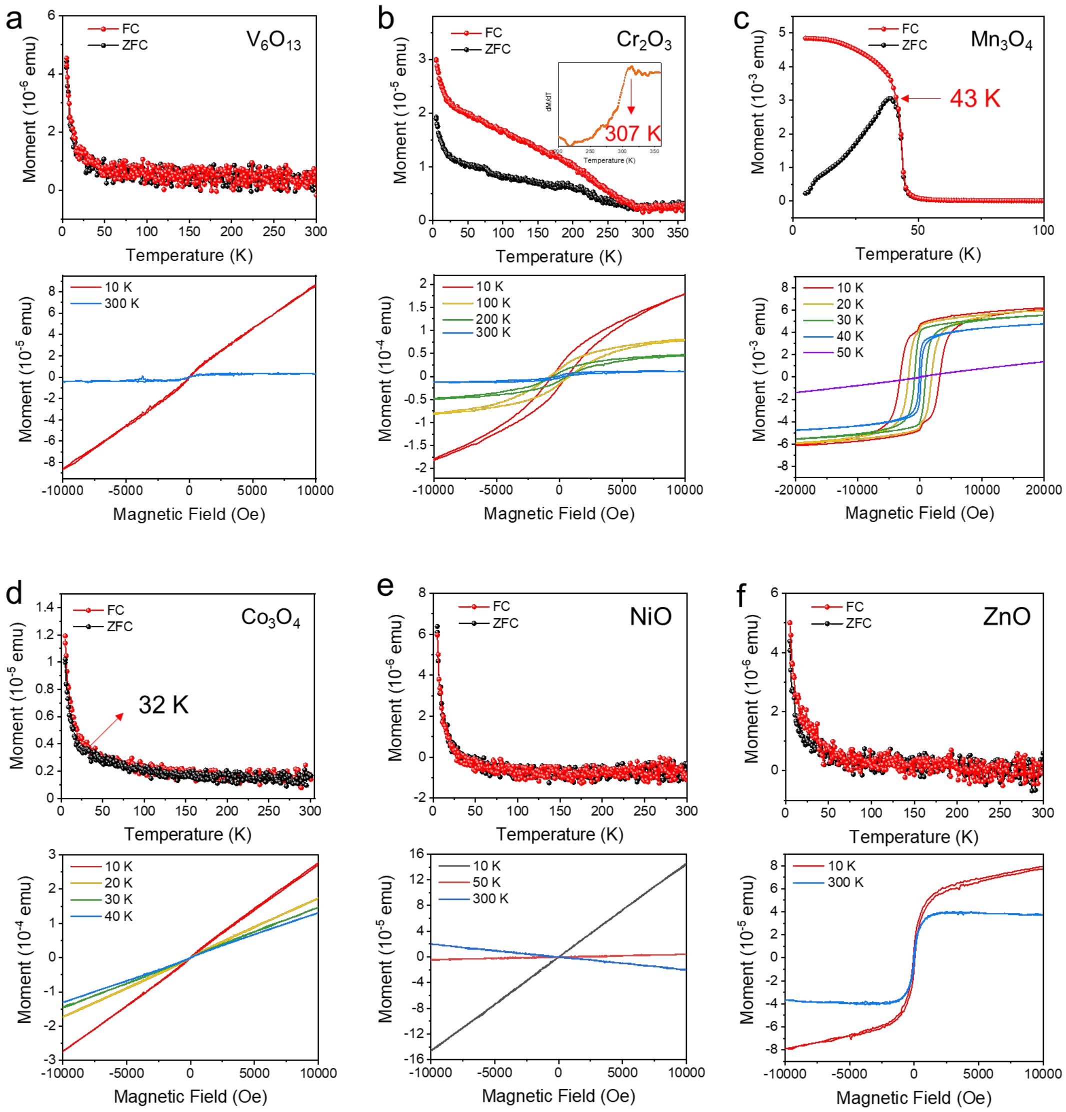

Supplementary Fig. 24. Magnetization curves of other binary oxides. a-f, Temperature-dependent magnetization curves (upper) and magnetic hysteresis curves (down) of V_6O_{13} (a), Cr_2O_3 (b), Mn_3O_4 (c), Co_3O_4 (d), NiO (e), and ZnO (f), respectively, with the magnetic field of 200 Oe parallel to the substrate.

V_6O_{13} is paramagnetic. Cr_2O_3 was reported to be antiferromagnetic with Neel temperature at ~307 K[8]. Mn_3O_4 shows Néel temperature at about 43 K with a large coercivity of ~3200 Oe at 10 K. The Néel temperature of Co_3O_4 is determined to be ~32 K. As for NiO, ZFC and FC curves exhibit paramagnetic-like behavior, because the Neel temperature is above room temperatures[9]. ZnO exhibits weak magnetism, which may originate from defects or strain.

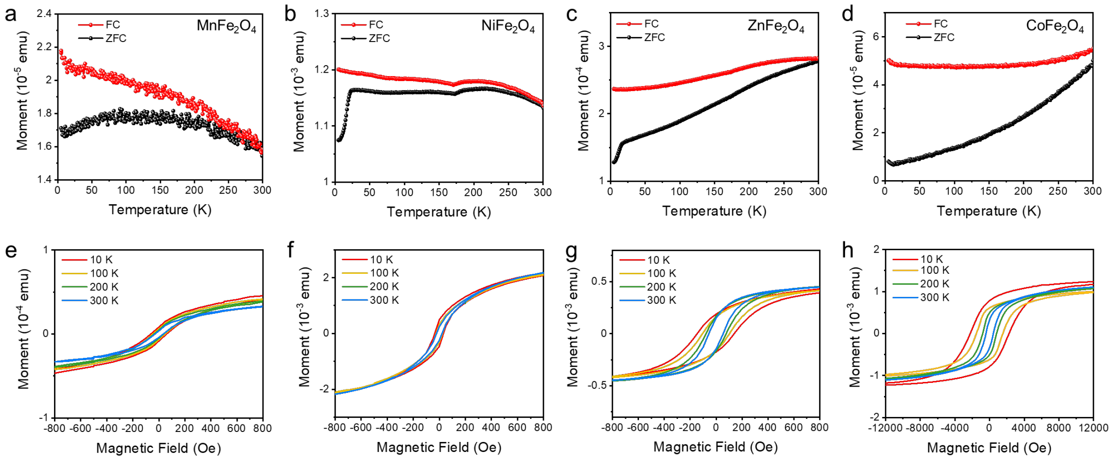

Supplementary Fig. 25. Magnetization curves of ternary oxides. a-d, Temperature-dependent magnetization curves of $MnFe_2O_4$ (a), $NiFe_2O_4$ (b), $ZnFe_2O_4$ (c), and $CoFe_2O_4$ (d), respectively, with the magnetic field of 200 Oe parallel to the substrate. **e-h,** Magnetization versus magnetic field curves at different temperatures after subtracting background signals at high fields of $MnFe_2O_4$ (e), $NiFe_2O_4$ (f), $ZnFe_2O_4$ (g), and $CoFe_2O_4$ (h), respectively.

All ternary ferrites exhibit room-temperature magnetism with different coercivity.

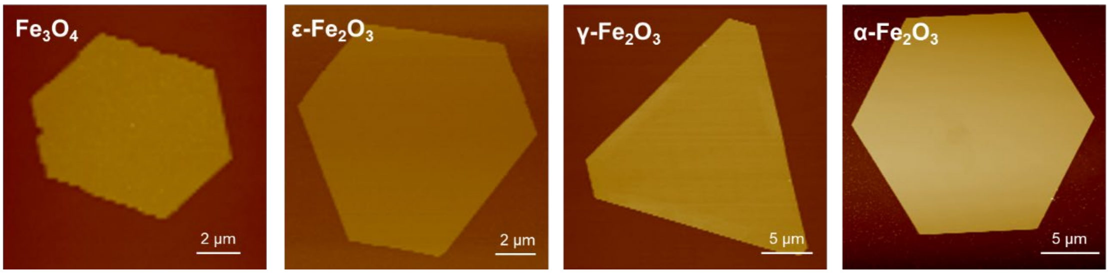

Supplementary Fig. 26. AFM surface topography of Fe-based oxides after exposure to air for three months.

There is no obvious oxidation in the images and the surface remains flat, indicating good stability of oxides.

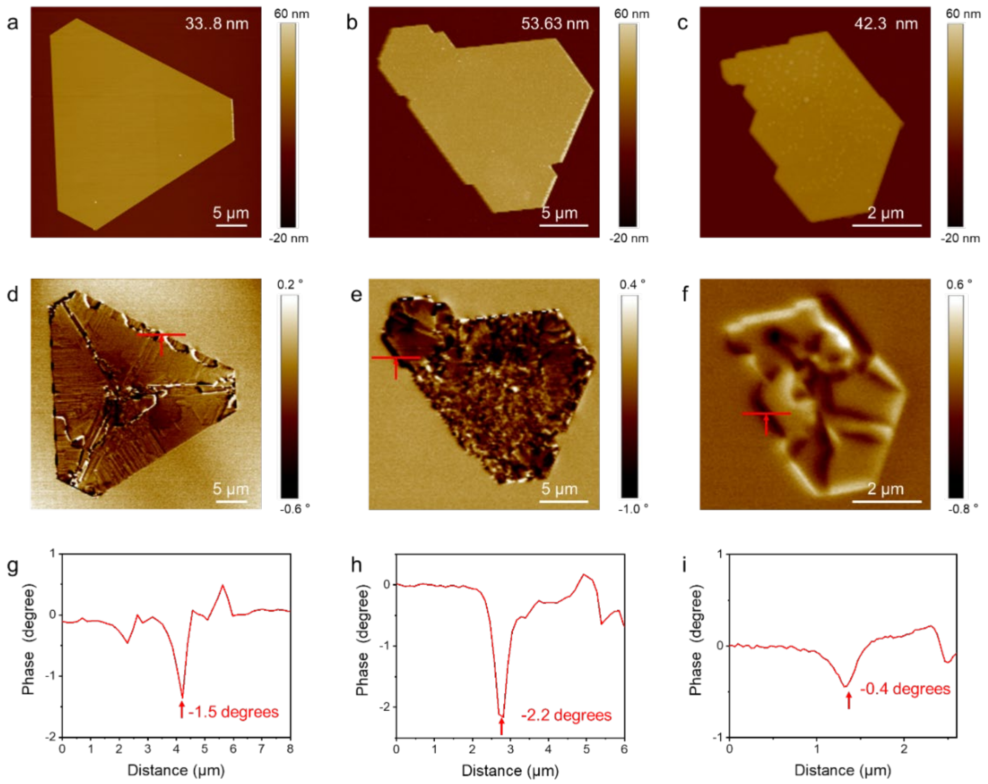

Supplementary Fig. 27. MFM images of Fe-based oxides with thicker thickness. a-c, Topological images of Fe_3O_4 (a), γ-Fe_2O_3 (b), and ε-Fe_2O_3 (c). **d-i**, MFM phase images and the corresponding line profiles across the red line of Fe_3O_4 (d, g), γ-Fe_2O_3 (e, h), and ε-Fe_2O_3 (f, i).

The response of MFM is mostly sensitive to the second derivative of the magnetostatic interaction between the sample and the magnetic tip. The magnetic moment of our MFM tip is normal to the nanoflake, so the MFM phase signal is proportional to out-of-plane stray magnetic field emanating from the surface of the sample. Therefore, the phase difference between materials and the substrate (nonmagnetic) reflects the strength of out-of-plane magnetism of the sample. In addition, the nonuniform phase contrast of the nanoflake indicates the multiple magnetic domain structures. As is shown in Fig. 5a, b and Supplementary Fig. 27d, e, Fe_3O_4 and γ-Fe_2O_3 have stronger phase contrast with the substrate (the color of the phase image is darker). Moreover, the phase difference between the material and substrate is larger in Fe_3O_4 (such as ~1.5 degrees across the line of Supplementary Fig. 27d) and γ-Fe_2O_3 (such as ~2.2 degrees across the line of Supplementary Fig. 27e). Therefore, Fe_3O_4 and γ-Fe_2O_3 show strong out-of-plane magnetism with different magnetic domain shapes. While ε-Fe_2O_3 has weaker contrast with the substrate (Supplementary Fig. 27f) and smaller phase differences with 0.4 degrees (Supplementary Fig. 27i) under similar thickness, indicating the in-plane magnetism. Moreover, several domains were formed by flux-closure or vortex-like structures in ε-Fe_2O_3.

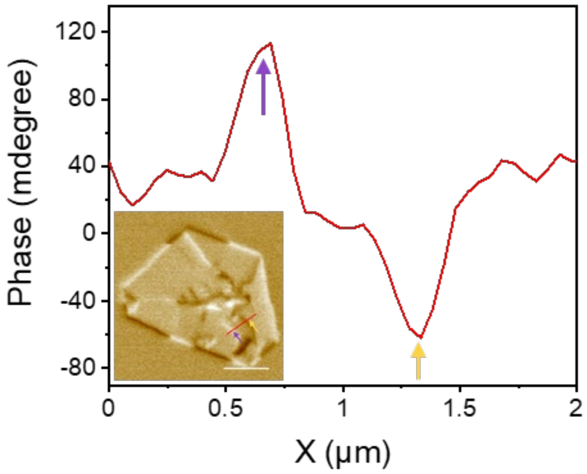

Supplementary Fig. 28. Line profile of ε-Fe_2O_3 MFM image.

MFM is sensitive to the out-of-plane stray field from the surface of the sample, so the weaker difference between ε-Fe_2O_3 and substrates with only ~40 mdegrees indicates the in-plane magnetism. On the other hand, the magnetic signal across the line profile exhibits double peaks, illustrating in-plane spin orientation as well[10].

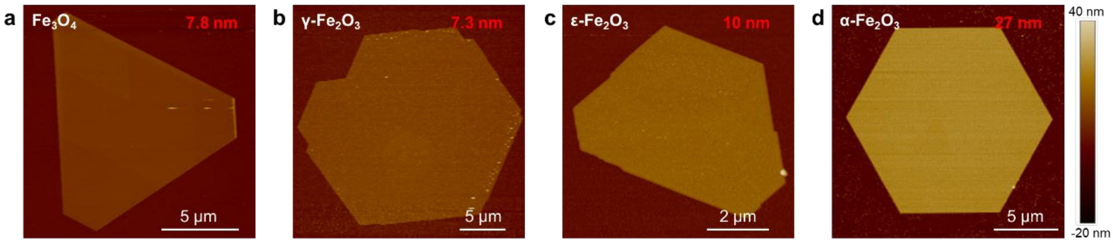

Supplementary Fig. 29. The MFM topography images of oxides.

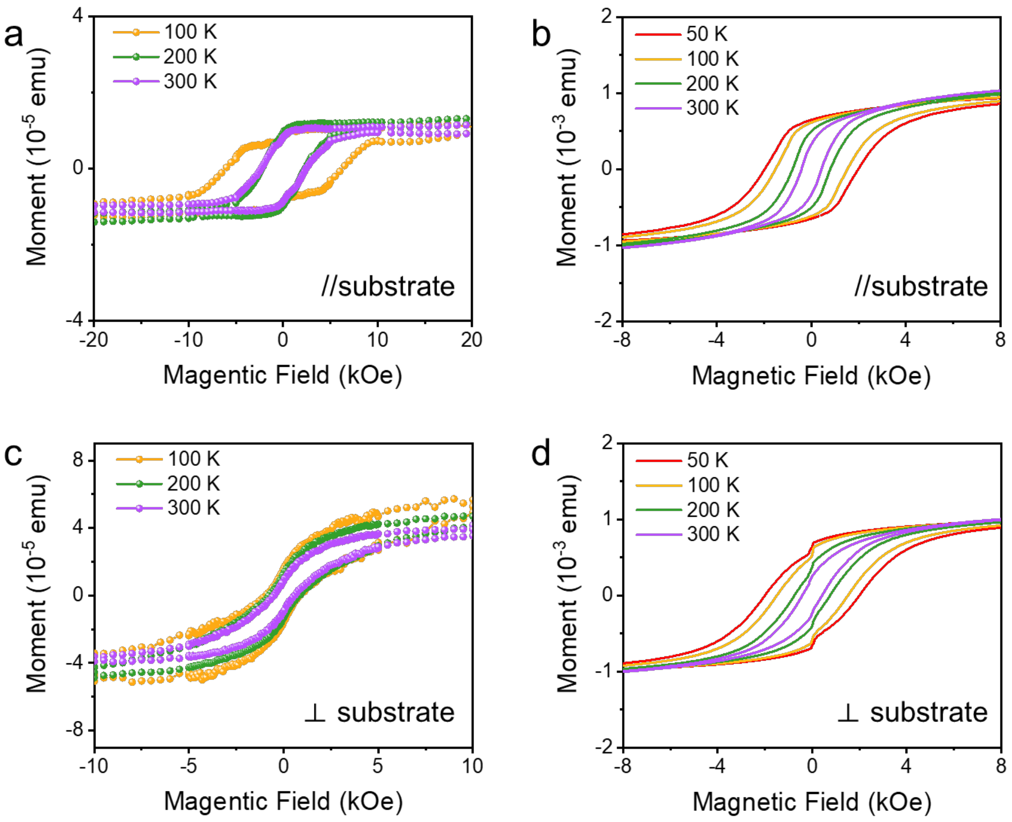

Supplementary Fig. 30. Magnetization curves of *ε*-Fe_2O_3 (a, c) and $CoFe_2O_4$ (b, d) under different magnetic field directions (after subtracting background signals at high fields).

As for *ε*-Fe_2O_3, when the magnetic field is parallel to the substrate, the loop is squarer and the coercivity is larger, indicating an in-plane easy-magnetization axis, which is in line with the MFM data as well. Besides, both strong in-plane and out-of-plane magnetism are detected in $CoFe_2O_4$.

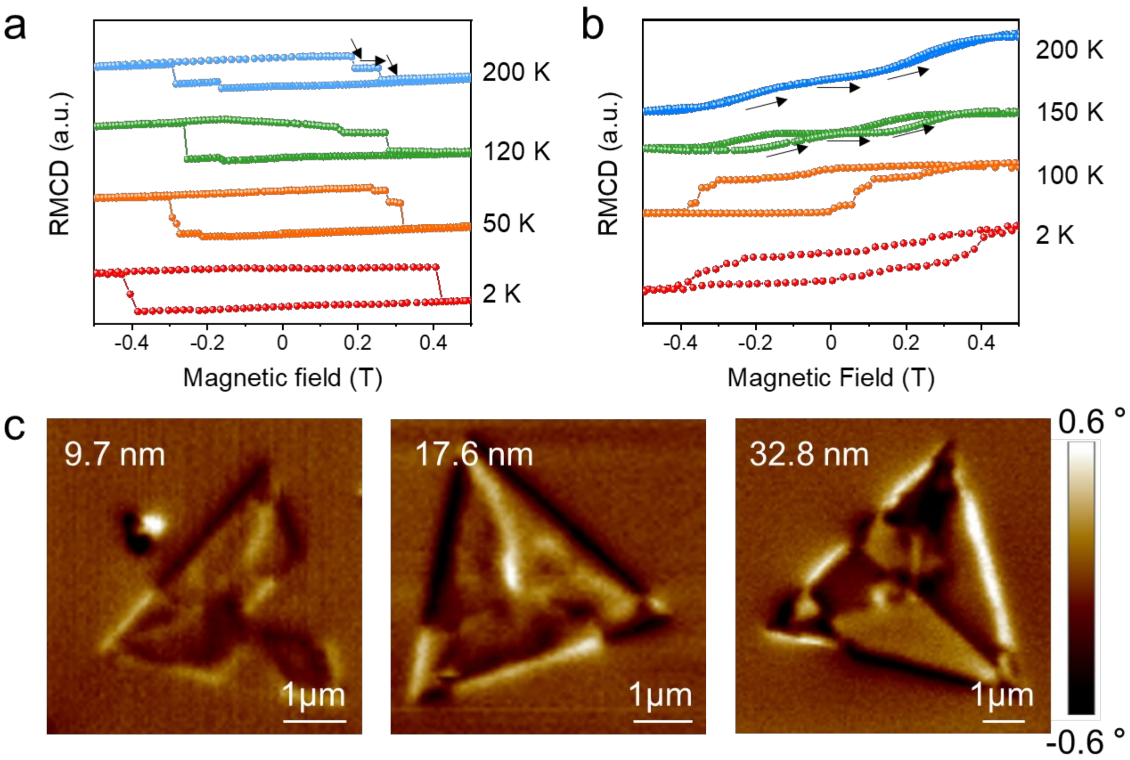

Supplementary Fig. 31. Thickness-dependent magnetic properties of $CoFe_2O_4$. a, RMCD signals of $CoFe_2O_4$ nanoflake with the thickness of ~60 nm. **b**, RMCD signals of $CoFe_2O_4$ nanoflake with the thickness of ~12 nm. **c**, Thickness-dependent MFM images of $CoFe_2O_4$ nanoflakes.

At thicker thickness (Supplementary Fig. 31a), RMCD signal shows a step-like loop (indicated by the arrows) as the magnetic field sweeps upward, and finally reaches the saturation value at a high positive field. Similar step-like hysteresis phenomena were also reported in 2D $CrTe_2$[11], CrI_3[12], and $CrBr_3$[13], which may derive from the polarization of several domains that are magnetically independent when the spot size of RMCD laser is ~1 μm. When the thickness is thinner down to ~12 nm (Supplementary Fig. 31b), step-like hysteresis is more obvious and the magnetic signal becomes weaker, illustrating the lowered magnetic exchange interaction. The large coercivity is well sustained with a little reduction (~3900 Oe for the thicker and ~3300 Oe for the thinner sample at 2 K). In addition, the multi-domain structure gets more sophisticated (the number of domain walls increases) and magnetism decreases (the phase contrast with the substrate becomes weaker) with reducing the thickness (Supplementary Fig. 31c), which may explain the step-like hysteresis behavior of RMCD results.

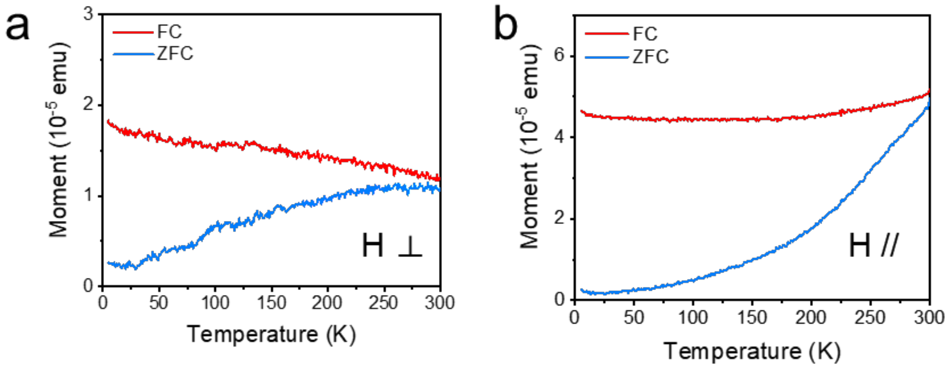

Supplementary Fig. 32. Temperature-dependent magnetization curves of $Mn_xFe_yCo_{3-x-y}O_4$ alloy. a, Magnetic field is perpendicular to the substrate. **b**, Magnetic field is parallel to the substrate.

The ZFC curves decrease gradually with the reduction of temperatures, so the spins are antiferromagnetic coupling. Moreover, the magnetic hysteresis is obvious even at 300 K in Fig. 5j, k, indicating the existence of uncompensated magnetic moment. Therefore, the alloy possesses ferrimagnetic behavior and room-temperature magnetism. Magnetism was detected in $Mn_xFe_yCo_{3-x-y}O_4$ nanoflakes regardless of applying a perpendicular or parallel magnetic field, which indicates that spins are canted with the existence of magnetic components in both in-plane and out-of-plane directions.

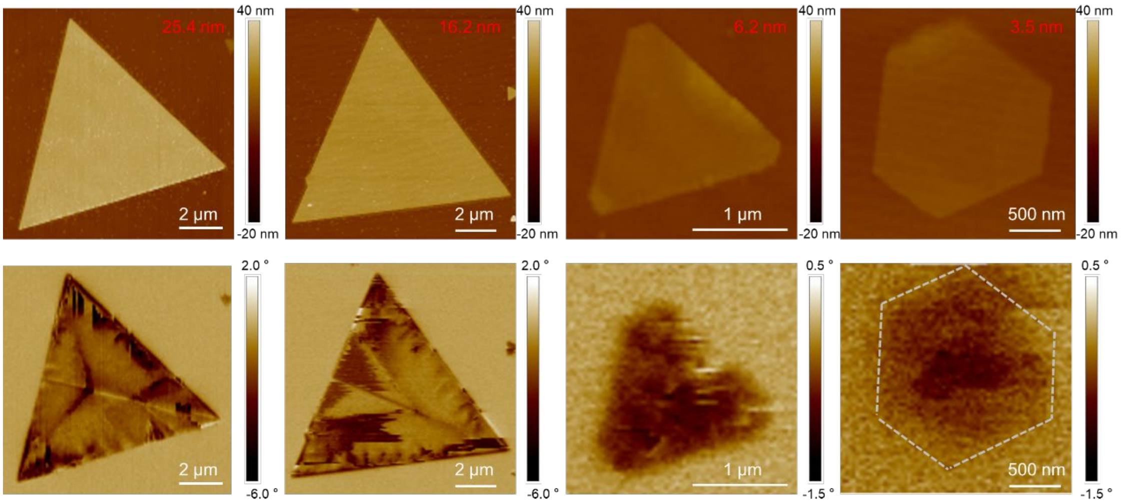

Supplementary Fig. 33. MFM images of $Mn_xFe_yCo_{3-x-y}O_4$ alloy with different thicknesses.

With the decrease of thickness, magnetic signals (phase contrasts) are well maintained even down to 3.5 nm (~2 unit cells), indicating strong room-temperature magnetism. Multi-domain magnetic states are also exhibited in all thicknesses. Besides, the magnetic domains have stronger phase contrast with the substrate (light and dark contrast sense is strong), indicating that $Mn_xFe_yCo_{3-x-y}O_4$ possesses out-of-plane magnetic vector.

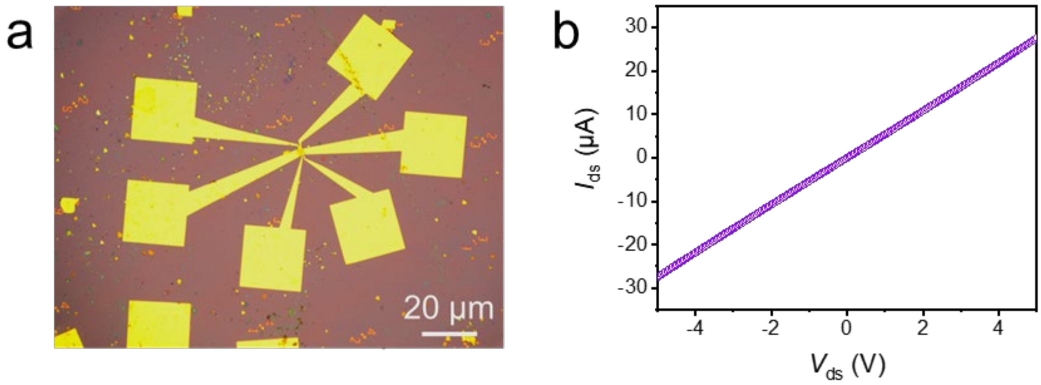

Supplementary Fig. 34. a. The optical image of Hall devices based on $Mn_xFe_yCo_{3-x-y}O_4$ alloy. b, Corresponding I_{ds}–V_{ds} curve of Hall devices.

The typical linear source-drain current (I_{ds}) versus source-drain voltage (V_{ds}) curve demonstrates the good ohmic contact between electrodes and nanoflakes.

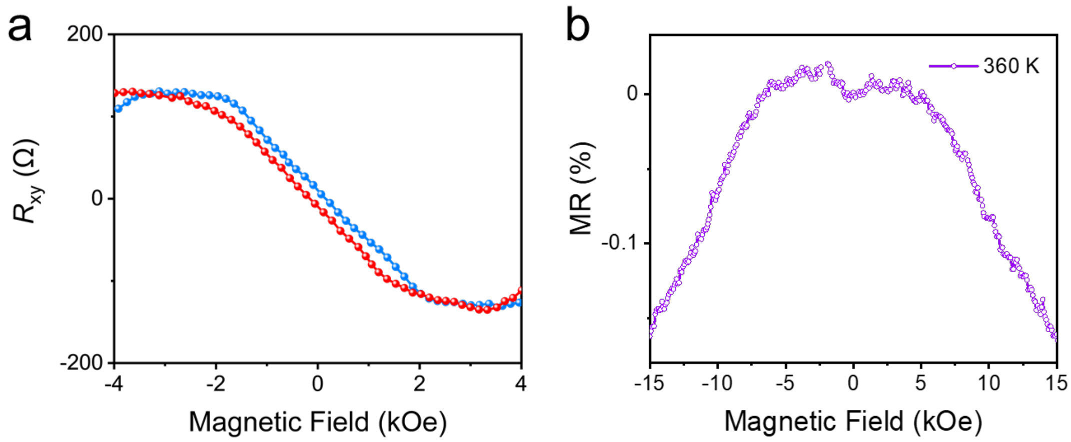

Supplementary Fig. 35. a, Magnetic field-dependent Hall resistance (R_{xy}) of $Mn_xFe_yCo_{3-x-y}O_4$ alloy at 360 K. Red dots are swept forward from the negative magnetic field to the positive magnetic field, and blue dots are swept backward. **b, Field-dependent magneto-resistance (MR) of $Mn_xFe_yCo_{3-x-y}O_4$ alloy at 360 K.**

The anomalous Hall effect (Supplementary Fig. 35a) and butterfly-shaped hysteresis phenomenon in the magneto-resistance curve (Supplementary Fig. 35b) indicate the appearance of magnetic ordering of $Mn_xFe_yCo_{3-x-y}O_4$ alloy even at 360 K.

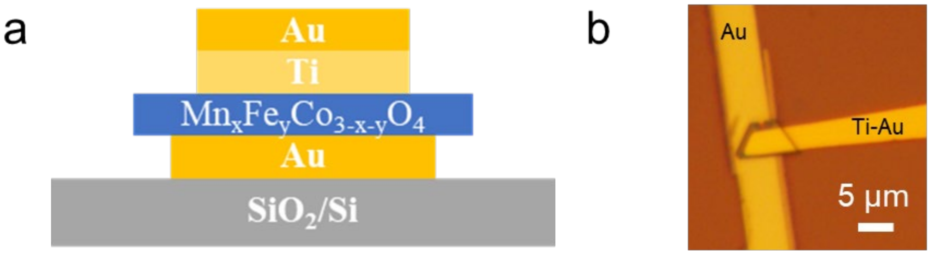

Supplementary Fig. 36. a, Schematic illustration of the vertical device with a sandwich Au/$Mn_xFe_yCo_{3-x-y}O_4$/Ti-Au structure. b, The optical image of the vertical device.

20 nm Au metals are deposited as the bottom electrode. Then, the nanoflakes are transferred on top of the bottom electrode via poly(methyl methacrylate)-assisted method. Next, the top electrode is defined by e-beam lithography and 5 nm Ti and 60 nm Au were deposited.

Supplementary Table 1. The differences between layered and nonlayered materials in our TTCG model.

	**Layered materials**	**Nonlayered materials**
Structures	Weak vdW binding	Strong covalent binding
Subunits	Generally represented by one slab between adjacent vdW gaps	The selection of subunit is more complex and needs to be discussed individually: different crystal orientations may have different subunits, and surface configurations also strongly affect the system energy (Supplementary Fig.1)
$\varepsilon_{i,i+1}$	Small (0.0145 eV/Å^2 for MoS_2)	Large (0.1578 eV/Å^2 for Fe_3O_4), about 10 times larger than layered materials
The relationship with *n*	$\varepsilon_{i,i+1}$ can be considered as a fixed value	$\varepsilon_{i,i+1}$ is related with n, as shown in Supplementary Figs. 2 and 3.

Supplementary Table 2. Diffusion barrier energy of different substrates.

Substrates	Diffusion barrier energy
Mica	0.1236 eV
Sapphire	0.8624 eV
Si (111)	1.2104 eV

The diffusion barrier energies of the adsorbate on the substrate are calculated via transition state search by nudged elastic band (NEB) method.

We assume that the influence of substrates on λ_c can be expressed as $\lambda_c = C \cdot Aexp(-E_{dif}/k_B T)$, where C, A, E_{dif}, k_B, and T are the energy conversion coefficient, diffusion constant, diffusion barrier energy, Boltzmann constant, and temperature, respectively. The energy conversion coefficient (C) indicates the influence of kinetic diffusion process on the edge binding interaction.

As is shown, mica has smaller diffusion barrier energy (E_{dif}), so their resistance for edge growth is small, especially at high temperatures (growth temperature is more than 800 K), leading to smaller λ_c to promote 2D growth. Therefore, we assume that λ_c can be considered as 0 for mica substrates in our work.

Supplementary Table 3. The structures of transition metal oxides.

Materials	Structures	Materials	Structures
Fe_3O_4	Cubic (Fd-3m)	Co_3O_4	Cubic (Fd-3m)
γ-Fe_2O_3	Cubic ($P4_132$)	NiO	Cubic (Fm-3m)
ε-Fe_2O_3	Orthogonal ($Pna2_1$)	ZnO	Trigonal ($P6_3mc$)
α-Fe_2O_3	Trigonal (R-3c)	$MnFe_2O_4$	Cubic (Fd-3m)
V_6O_{13}	Monoclinic ($P2_1/c$)	$CoFe_2O_4$	Cubic (Fd-3m)
Cr_2O_3	Trigonal (R-3c)	$NiFe_2O_4$	Cubic (Fd-3m)
Mn_3O_4	Tetragonal ($I4_1/amd$)	$ZnFe_2O_4$	Cubic (Fd-3m)

Supplementary Table 4. **The calculated energies based on the model (in the unit of eV/Å^2).**

Structure	$\varepsilon_{n,n+1}$	Correction term ε_c of water	λ_n+λ_B	ΔE without correction	ΔE with correction
Fe_3O_4-111	0.1578	0.3749	0.2692	-0.1114	-0.4863
Fe_3O_4-001	0.1138	0.2650	0.2038	-0.0900	-0.3550
Fe_3O_4-110	0.0576	0.0662	0.2698	-0.2122	-0.2784
γ-Fe_2O_3-111	0.2600	0.0919	0.6655	-0.4055	-0.4974
γ-Fe_2O_3-001	0.3761	0.1341	0.6343	-0.2582	-0.3923
γ-Fe_2O_3-110	0.3171	0.1004	0.6655	-0.3484	-0.4488
α-Fe_2O_3-111	0.0358	0.0703	0.2806	-0.2448	-0.3151
α-Fe_2O_3-001	0.0310	0.0894	0.2772	-0.2462	-0.3356
α-Fe_2O_3-110	0.0201	0.0580	0.2806	-0.2605	-0.3185
ε-Fe_2O_3-111	0.0856	0.0684	0.2840	-0.1984	-0.2668
ε-Fe_2O_3-001	0.1983	0.1474	0.349	-0.1507	-0.2981
ε-Fe_2O_3-110	0.1589	0.0948	0.284	-0.1251	-0.2199
ZnO-001	0.6672	0.0663	0.1856	0.4816	0.4153
$CoFe_2O_4$-111	0.2686	0.2850	0.5154	-0.2468	-0.5318
$CoFe_2O_4$-001	0.2209	0.2127	0.4437	-0.2228	-0.4355
$CoFe_2O_4$-110	0.2563	0.1854	0.5154	-0.2591	-0.4445
EuOCl-001	0.0144	/	0.4819	-0.4675	/
SmOCl-001	0.0481	/	0.4936	-0.4455	/
Fe_7S_8-001	0.1842	/	0.4643	-0.2801	/

Cr_5Te_8-100	0.1308	/	0.3899	-0.2591	/
MoS_2	0.0145	/	0.7473	-0.7328	/
In_2Se_3-001	0.0708	/	0.2508	-0.1800	/
In_2Se_3-111	0.1225	/	0.2506	-0.1281	/
In_2Se_3-110	0.0820	/	0.2506	-0.1686	/
InSe	0.0260	/	0.2696	-0.2436	/

1) Detailed calculation procedure of the above parameters:

$\varepsilon_{n,n+1}$ is calculated by the formula $\varepsilon_{n,n+1} = (E_{n+1} - E_n)/A$, where the E_n and E_{n+1} are the total free energy of slabs with *n* and $n+1$ subunits, respectively. Therefore, $\varepsilon_{n,n+1}$ represents the binding energy between the subunit *n* and subunit $n+1$.

λ_n refer to the average edge energy of the initial system with *n* subunit. The total energy of edge plane is defined as E_{edge}. Then, λ_n is calculated by $\lambda_n = \frac{1}{k}\sum_{i=1}^{k}\left[(E_{edge} - N \cdot E_{unit})/2A\right]$, where E_{unit} and *N* are the energy of one unit cell and the number of unit cells in the slab. *i*=1~*k* represents different edge planes and λ_n is the average energy of these edge planes. Similarity, λ_B refer to the average edge energy of the new growth cluster and the calculation method is the same as that of λ_n. In the same material, we suppose λ_B equals λ_n.

ε_c is the correction term of ε term. The interface adsorption will passivate the dangling bonds of the top surface, thus reducing the binding energy between the initial structure and the newly growth cluster. ε_c of water is calculated by the formula $\varepsilon_c = E_{slab+H_2O} - E_{slab} - n \cdot E_{H_2O}$, where E_{slab+H_2O}, E_{slab} are the total free energies of the slab with and without adsorbed H_2O, respectively. E_{H_2O} and *n* are the energies of one H_2O molecular and the number of adsorbed H_2O on metal sites, respectively.

$\Delta E = (\varepsilon_{n,n+1} - \varepsilon_c)A_{s_B} - (\lambda_n + \lambda_B - \lambda_c)A_{l_B}$, where λ_c is 0 for mica substrate (Supplementary Table 2). A_{s_B} and A_{l_B} of the new growth cluster are assumed to be the same (supposed as unit area).

2) Notes:

The smaller the value of ΔE, the easier it tends to grow into 2D structures. We assume that when $\Delta E > 0$, 2D growth can not be realized. When -0.25 eV/Å^2< ΔE <0, 2D growth is more difficult. When ΔE < -0.25 eV/Å, it is inclined to grow 2D nanoflakes.

As for Fe-based oxides, the intrinsic energy differences between $\varepsilon_{n,n+1}$ and λ_n+λ_B are not negative enough (most of them >-0.25 eV/Å^2). The facilitation of H_2O passivation (large ε_c) and mica substrate (small λ_c, Supplementary Table 2) lead to smaller ΔE (<-0.3 eV/Å^2) after correction, and therefore promote the synthesis of ultrathin nanoflakes.

The calculated ΔE of ZnO is positive with 0.48 eV/Å^2 (without correction) or 0.42 eV/Å^2 (with correction), so it is unfavorable for 2D growth.

Nonlayered rare-earth metal oxyhalides (MOCl, M= rare metal) have a quasi-layered structure by alternating $[MO]^+$ and $[Cl]^-$ layers along the *c*-axis[14], so the $\varepsilon_{n,n+1}$ is much weaker than λ_n, leading to negative ΔE along the [001] direction. Taking EuOCl and SmOCl as examples, the intrinsic ΔE is indeed smaller, in favor of forming 2D nanoflakes. Besides, some transition metal chalcogens (such as Cr_5Te_8 and Fe_7S_8) possess metal vacancies along fixed directions[15], which reduces $\varepsilon_{n,n+1}$ interaction to have a good potential for 2D growth. Therefore, their intrinsic structural properties provide the possibility for growing into 2D structures to some extent.

γ-In_2Se_3 is in a nonlayered hexagonal structure. The calculated results show that it prefers to form 2D nanoflakes along the [001] direction with the ΔE of -0.18 eV/Å^2. The value is negative but not particularly small, so it may form 2D structure, but the formation of ultrathin thickness without other assistance may be difficult[16].

Supplementary Table 5. Principal lattice parameters and formation energy of an oxygen vacancy in iron oxides.

Phase	Calculated lattice parameter (Å)	E_{form} (eV/f.u.)
ε-Fe_2O_3	a = 5.189<br>b = 8.920<br>c = 9.567	0.422
γ-Fe_2O_3	a = b = c = 8.526	0.152
α-Fe_2O_3	a = b = 5.123<br>c = 13.873	0.470

In order to elucidate the phase transition under oxygen (O) deficiency, we employ first principles density functional theory (DFT) calculation with its exchange correlation functional treated by the generalized gradient approximation. The formation energy of an O vacancy[17,18] is computed via

$$E_{form} = \left[E_{V_O} - E_0\right] + \mu_O$$

Here, E_0 and E_{vanc} are the total energy of a pristine structure and with an O vacancy, respectively. Each supercell contains at least six formula units. μ_O is the chemical potential of oxygen. The principal lattice parameters and the calculated formation energies are listed in the Table. Larger E_{form} indicate poorer stability in the oxygen-deficient environment. Thus, γ-Fe_2O_3 is favorable at low oxygen content (O/Fe ratio), and α-Fe_2O_3 is favorable at high oxygen content (O/Fe ratio). Obviously, Fe_3O_4 is formed when the O/Fe ratio is far less than 3/2.

Therefore, the DFT calculations reveal that the stability of Fe_3O_4, γ-Fe_2O_3, ε-Fe_2O_3, and α-Fe_2O_3 gradually reduces in the oxygen-deficient environment, qualitatively consistent with our experimental results (Supplementary Fig. 6).

Supplementary Table 6. The location of Raman peaks and corresponding vibration modes of Fe_3O_4, γ-Fe_2O_3, ε-Fe_2O_3, and α-Fe_2O_3.

Materials	Raman peaks	Raman modes	Materials	Raman peaks	Raman modes
**Fe_3O_4**	196	T_{2g} (1)	**ε-Fe_2O_3**	121	M1
	308	E_g		149	M2
	545	T_{2g} (2)		172	M3
	668	A_{1g}		196	M4
				236	M5
				267	M6
**γ-Fe_2O_3**	115	T_{2g}		309	M7
	262	T_{2g}		325	M8
	330-350	T_{2g}		354-375	M9- M11
	480-530	E_g		395	M12
	650-720	A_{1g}		420-460	M13- M15
	~1200	magnon scattering		485	M16
	~1430	magnon scattering		508	M17
				542	M18
				573	M19
**α-Fe_2O_3**	225	A_{1g}		645	M20
	245	E_g		685	M21
	297	E_g		700-850	M22- M24
	411	E_g		1120	M25
	497	A_{1g}		~1350	M26- M28
	611	E_g		~1450	M29- M30
	1320	magnon scattering		1600	M31

Supplementary Table 7. The comparison of the sizes and qualities between as-synthesized iron oxides samples and other literatures.

	**The largest size in the reference**	**The thinnest thickness in the reference**	**The largest size in this work**	**The thinnest thickness in this work**
Fe_3O_4	2–4 μm[19]	1.95 nm[19]	40 μm	6.1 nm
γ-Fe_2O_3	Non-uniform surface with impurities [20]	4.61 nm[20]	44 μm	5.4 nm
ε-Fe_2O_3	~20 μm[21]	5.1 nm[21]	133 μm	5.1 nm

Supplementary Table 8. The summary of magnetism in 2D oxides nanoflakes.

Materials	Magnetism	Critical temperatures
Fe_3O_4	Ferrimagnetic (FiM)	**>300 K**<br>(Verwey transition at 130 K)
γ-Fe_2O_3	FiM	**>300 K**
ε-Fe_2O_3	FiM<br>Hard magnets	**>300 K**<br>(Metamagnetization at 150 K)
α-Fe_2O_3	Antiferromagnetic<br>(AFM)	**>300 K**
Cr_2O_3	AFM	**~307 K**
Mn_3O_4	AFM<br>Hard magnets	~43 K
Co_3O_4	AFM	~32 K
NiO	AFM	**>300 K**
ZnO	Weak FM	/
V_6O_{13}	Paramagnetic	/
$MnFe_2O_4$	FiM	**>300 K**
$CoFe_2O_4$	FiM<br>Hard magnets	**>300 K**
$NiFe_2$O4	FiM	**>300 K**
$ZnFe_2O_4$	FiM	**>300 K**

Supplementary Table 9. The synthesis recipes for transition-metal-based oxides

Materials	Reactant	Temperatures	Atmosphere (100 sccm Ar)
Fe_3O_4	15 mg $FeCl_2$	600 °C	0 sccm O_2
γ-Fe_2O_3	15 mg $FeCl_2$	600 °C	1 sccm O_2 60 s
ε-Fe_2O_3	15 mg $FeCl_2$	600 °C	1 sccm O_2 150 s
α-Fe_2O_3	15 mg $FeCl_2$	600 °C	1 sccm O_2 600 s
V_6O_{13}	15 mg VCl_3	600 °C	0 sccm O_2
Cr_2O_3	10 mg $CrCl_2$	850 °C	1 sccm O_2
Mn_3O_4	40mg $MnCl_2$	680 °C	0 sccm O_2
Co_3O_4	15 mg $CoCl_2$	880 °C	1 sccm O_2
NiO	15 mg $NiCl_2$	750 °C	0 sccm O_2
ZnO	30 mg $ZnCl_2$	550 °C	0 sccm O_2
$MnFe_2O_4$	20 mg $FeCl_2$+10 mg $MnCl_2$	650 °C	1 sccm O_2 60 s
$CoFe_2O_4$	20 mg $FeCl_2$+20 mg $CoCl_2$	680 °C	1 sccm O_2 60 s
$NiFe_2O_4$	10 mg $FeCl_2$+20 mg $NiCl_2$	750 °C	1 sccm O_2 60 s
$ZnFe_2O_4$	15 mg $FeCl_2$+15 mg $ZnCl_2$	550 °C	1 sccm O_2 60 s
Alloy	corresponding chloride precursor	700 °C	1 sccm O_2 60 s

Supplementary Note 1: Detailed deducing process of the equations (2) and (3)

We assume a new growth cluster (the basal and lateral area are A_{S_B} and A_{L_B}, the number of superimposed subunits is *m*) combines with the initial structure (the basal and lateral area are A_S and A_L, the number of superimposed subunits is *n*) in two ways, *i.e.*, vertically or laterally. According to the equation (1), the total free energy of the initial structure (E_{free}^{n}) and the newly growth cluster (E_{free}^{m}) is as follow:

$$E_{free}^{n} = \sum_{i=1}^{n} E_i A_S - \sum_{i=1}^{n-1} \varepsilon_{i,i+1} A_S + \sum_{i=1}^{n} \lambda_i A_L$$

$$E_{free}^{m} = \sum_{i=1}^{m} E_i A_{S_B} - \sum_{i=1}^{m-1} \varepsilon_{i,i+1} A_{S_B} + \sum_{i=1}^{m} \lambda_i A_{L_B}$$

After vertical growth, the binding energy of the subunit at the interface will change, and the total free energy is as follows.

$$E_{ver} = (\sum_{i=1}^{n} E_i A_S + \sum_{i=1}^{m} E_i A_{S_B}) - (\sum_{i=1}^{n-1} \varepsilon_{i,i+1} A_S + \sum_{i=\mathrm{n}}^{m+\mathrm{n}-1} \varepsilon_{i,i+1} A_{S_B}) + (\sum_{i=1}^{n} \lambda_i A_L + \sum_{i=1}^{m} \lambda_i A_{L_B})$$

We suppose that the change of $\varepsilon_{i,i+1}$ (influenced by *n*) can be ignored when *n* is larger than 4 (as shown in Supplementary Fig. 3). The basal contact area A_{S_B} equals A_{S_B}. Therefore,

$$\Delta E_{ver} = E_{ver} - E_{free}^{n} - E_{free}^{m} = -(\sum_{i=\mathrm{n}}^{m+\mathrm{n}-1} \varepsilon_{i,i+1} - \sum_{i=1}^{m-1} \varepsilon_{i,i+1}) A_{S_B} = -\varepsilon_{n,n+1} A_{S_B}$$

After lateral growth, we define the lateral contact area is A_{l_B}. The edge energy is decreased due to the reduced lateral area, and the total free energy is as follows.

$$E_{lat} = (\sum_{i=1}^{n} E_i A_S + \sum_{i=1}^{m} E_i A_{S_B}) - (\sum_{i=1}^{n-1} \varepsilon_{i,i+1} A_S + \sum_{i=1}^{m-1} \varepsilon_{i,i+1} A_{S_B}) + (\sum_{i=1}^{n} \lambda_i A_L - \lambda_n A_{l_B} + \sum_{i=1}^{m} \lambda_i A_{L_B} - \lambda_B A_{l_B})$$

where λ_n and λ_B represent the average edge energies of the initial structure and the new cluster, respectively.

Therefore,

$$\Delta E_{lat} = E_{lat} - E_{free}^{n} - E_{free}^{m} = -\lambda_n A_{l_B} - \lambda_B A_{l_B}$$

Supplementary References

1. Wen, Y. et al. Tunable Room-Temperature Ferromagnetism in Two-Dimensional Cr_2Te_3. *Nano Lett.* **20**, 3130-3139 (2020).
2. Becker, M. et al. Phase Control of Multivalent Vanadium Oxides VOx by Ion-Beam Sputter-Deposition. *Phys. Status Solidi A* **219**, 2100828 (2022).
3. Wang, P. et al. Controllable Synthesis Quadratic-Dependent Unsaturated Magnetoresistance of Two-Dimensional Nonlayered Fe_7S_8 with Robust Environmental Stability. *ACS Nano* **16**, 8301-8308 (2022).
4. Tang, B. et al. Phase engineering of Cr_5Te_8 with colossal anomalous Hall effect. *Nat. Electron.* **5**, 224-232 (2022).
5. Martín-García, L. et al. Spin reorientation transition of magnetite (001). *Phys. Rev. B* **93**, 134419 (2016).
6. Gich, M., Frontera, C., A. Roig, E. T. & Molins, E. High- and Low-Temperature Crystal and Magnetic Structures of E-Fe_2O_3 and Their Correlation to Its Magnetic Properties. *Chem. Mater.* **18**, 3889-3897 (2006).
7. Puthirath Balan, A. et al. Exfoliation of a non-van der Waals material from iron ore hematite. *Nat. Nanotechnol.* **13**, 602-609 (2018).
8. Shiratsuchi, Y., Nakatani, T., Kawahara, S.-i. & Nakatani, R. Magnetic coupling at interface of ultrathin Co film and antiferromagnetic Cr_2O_3 (0001) film. *J. Appl. Phys.* **106**, 033903 (2009).
9. Popkov, S. I. et al. Size effects in the formation of an uncompensated ferromagnetic moment in NiO nanoparticles. *J. Appl. Phys.* **126**, 103904 (2019).
10. Geng, Y., Lee, N., Choi, Y. J., Cheong, S. W. & Wu, W. Collective Magnetism at Multiferroic Vortex Domain Walls. *Nano Lett.* **12**, 6055-6059 (2012).
11. Purbawati, A. et al. In-Plane Magnetic Domains and Neel-like Domain Walls in Thin Flakes of the Room Temperature $CrTe_2$ Van der Waals Ferromagnet. *ACS Appl. Mater. Interfaces* **12**, 30702-30710 (2020).
12. Huang, B. et al. Layer-dependent ferromagnetism in a van der Waals crystal down to the monolayer limit. *Nature* **546**, 270-273 (2017).
13. Zhang, Z. et al. Direct Photoluminescence Probing of Ferromagnetism in Monolayer Two-Dimensional $CrBr_3$. *Nano Lett.* **19**, 3138-3142 (2019).
14. Berdowski, P. A. M., HerkJ., V. & Blasse, G. Energy migration in a quasi-two-

dimensional system: EuOCl. *J. Lumin.* **34**, 9-18 (1985).

15. Li, F. & Franzen, H. Ordering, Incommensuration, and Phase Transitions in Pyrrhotite. Part I: A TEM Study of Fe_7S_8. *J. Solid State Chem.* **124**, 264-271 (1996).

16. Huang, W. et al. Chemical vapor deposition growth of nonlayered γ-In_2Se_3 nanosheets on SiO_2/Si substrates and its photodetector application. *J. Alloys Compd.* **904**, 164010 (2022).

17. Cheng, Y. et al. Vacancy formation energy and its connection with bonding environment in solid: A high-throughput calculation and machine learning study. *Comp. Mater. Sci.* **183**, 109803 (2020).

18. Edwards, A. et al. Electronic structure of intrinsic defects in crystalline germanium telluride. *Phys. Rev. B* **73**, 045210 (2006).

19. Yin, C. et al. Ultrabroadband Photodetectors up to 10.6 μm Based on 2D Fe_3O_4 Nanosheets. *Adv. Mater.* **32**, 2002237 (2020).

20. Jia, Z. et al. Morphology-Tunable Synthesis of Intrinsic Room-Temperature Ferromagnetic γ-Fe_2O_3 Nanoflakes. *ACS Appl. Mater. Interfaces* **13**, 24051-24061 (2021).

21. Yuan, J. et al. Room-Temperature Magnetic Order in Air-Stable Ultrathin Iron Oxide. *Nano Lett.* **19**, 3777-3781 (2019).
